# High Concentrations of Perfluoroalkyl Acids in Arctic Seawater Driven by Early Thawing Sea Ice

**DOI:** 10.1021/acs.est.1c01676

**Published:** 2021-07-26

**Authors:** Jack Garnett, Crispin Halsall, Anna Vader, Hanna Joerss, Ralf Ebinghaus, Amber Leeson, Peter M. Wynn

**Affiliations:** † Lancaster Environment Centre, Lancaster University, Lancaster LA1 4YQ, U.K.; ‡ Department of Arctic Biology, The University Centre in Svalbard (UNIS), Longyearbyen N-9170, Norway; § Helmholtz-Zentrum Hereon, Max-Planck-Straße 1, Geesthacht 21502, Germany

**Keywords:** PFAS, Arctic, sea ice, seawater, snow, meltpond, chemical exposure

## Abstract

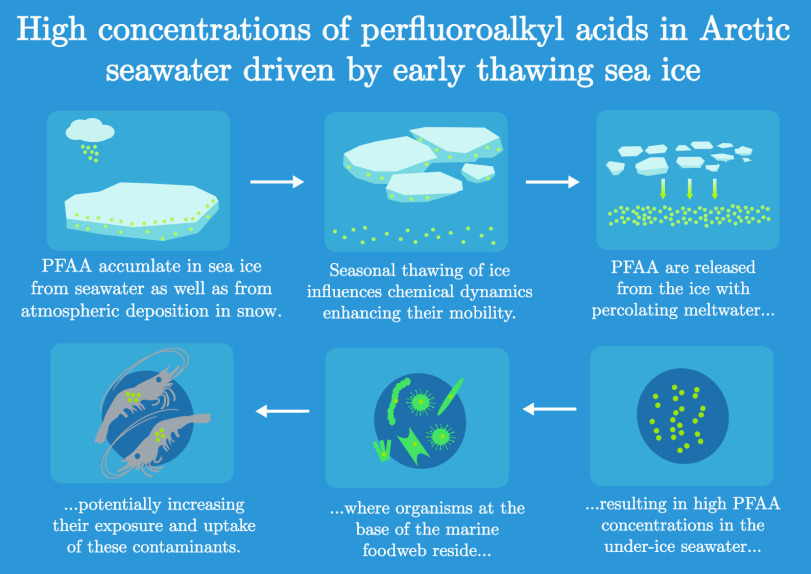

Poly- and perfluoroalkyl substances are synthetic chemicals that are widely present in the global environment including the Arctic. However, little is known about how these chemicals (particularly perfluoroalkyl acids, PFAA) enter the Arctic marine system and cycle between seawater and sea ice compartments. To evaluate this, we analyzed sea ice, snow, melt ponds, and near-surface seawater at two ice-covered stations located north of the Barents Sea (81 °N) with the aim of investigating PFAA dynamics in the late-season ice pack. Sea ice showed high concentrations of PFAA particularly at the surface with snow-ice (the uppermost sea ice layer strongly influenced by snow) comprising 26–62% of the total PFAA burden. Low salinities (<2.5 ppt) and low δ^18^O_H20_ values (<1‰ in snow and upper ice layers) in sea ice revealed the strong influence of meteoric water on sea ice, thus indicating a significant atmospheric source of PFAA with subsequent transfer down the sea ice column in meltwater. Importantly, the under-ice seawater (0.5 m depth) displayed some of the highest concentrations notably for the long-chain PFAA (e.g., PFOA 928 ± 617 pg L^–1^), which were ≈3-fold higher than those of deeper water (5 m depth) and ≈2-fold higher than those recently measured in surface waters of the North Sea infuenced by industrial inputs of PFAAs. The evidence provided here suggests that meltwater arising early in the melt season from snow and other surface ice floe components drives the higher PFAA concentrations observed in under-ice seawater, which could in turn influence the timing and extent of PFAA exposure for organisms at the base of the marine food web.

## Abstract 2

Sea ice accumulates perfluoroalkyl acids (PFAAs) from seawater and from atmospheric deposition in snow. Subsequent melting of surface ice layers through seasonal changes later results in high PFAA concentrations in the under-ice seawater.

## 1 Introduction

Poly- and perfluoroalkyl substances (PFASs) consist of a large group of synthetic chemicals that are used in a wide variety of industrial and consumer applications.^1^ Perfluoroalkyl acids (PFAAs), including the perfluoroalkyl carboxylic acids (PFCAs) and perfluoroalkyl sulfonic acids (PFSAs), are a major group of PFASs, which have a ubiquitous presence in the global environment. Moreover, long-chain PFAAs (long chain = PFCA with eight carbons and greater and PFSA with six carbons and greater^2^) are bioaccumulative and display a range of adverse toxic effects in humans and biota. Understanding the fate and behavior of PFAAs in the environment is therefore important particularly in relation to remote ecosystems such as the Arctic that are reported to be currently experiencing other environmental stressors.^3^

PFAAs have been observed in the remote Greenland Sea^4^ and Chukchi/Beaufort Sea regions of the western Arctic^5^ with their presence having been linked to transport through ocean currents originating from industrial regions. PFAAs are also transported to remote environments such as the Arctic indirectly through photochemical oxidation of volatile precursors in the atmosphere followed by deposition, with their occurrence in the central Arctic Basin snowpack^5^ and on the Devon Island ice cap^6−8^ as evidence of atmospheric deposition. However, little is known about the relative importance of these two pathways with even less information about the fate and behavior of PFAS in sea ice and their subsequent fate during seasonal thaw.

The observation of PFAAs in sea ice along with other persistent organic pollutants is limited to a handful of field studies,^5,9−14^ but only recent mechanistic investigations have revealed that brine in sea ice plays an important role in distributing these chemicals during sea ice growth.^10,15^ Sea ice brine has also been shown to contain contaminant concentrations at levels significantly greater than those observed in the underlying seawater.^10,15^ In the warming Arctic Ocean dominated by brine-rich single-season ice, this has important implications for contaminant exposure to the many organisms situated at the base of the pelagic food web, which are abundant in sea ice. Sympagic organisms, such as ice algae and associated heterotrophic protists and metazoans, inhabit a network of brine inclusions and brine channels at the base of the ice and may be particularly vulnerable to brine which is enriched in contaminants.^13^

The late-season ice pack is a dynamic system, whereby organic contaminants that have accumulated in the winter snowpack are revolatilized to the atmosphere^16^ or transferred in meltwater to deeper layers of the sea ice system^14^ or even pass directly into the under-ice seawater. Melt ponds are also common features on late-season ice floes (Fetterer and Untersteiner, 1998),^100^ and both snowfall/precipitation and gas exchange with the overlying atmosphere have been shown to increase some contaminant levels in the ponds.^17^ Due to their high water solubilities and low volatility, PFAAs are likely to be transferred from thawing snow and sea ice to seawater. The aim of this study was to determine the concentrations and distribution of PFAAs in the various compartments of the late-season sea ice system and investigate the fate of PFAAs during the thawing process. Physical and chemical properties of snow, sea ice, and seawater including density, salinity, and stable isotope analysis were measured to evaluate the quantity of entrained meteoric water in ice/meltwater components and the role of melt/freshwater in PFAA fate.

## 2 Methods

### 2.1 Sample Collection

**Figure 1:**
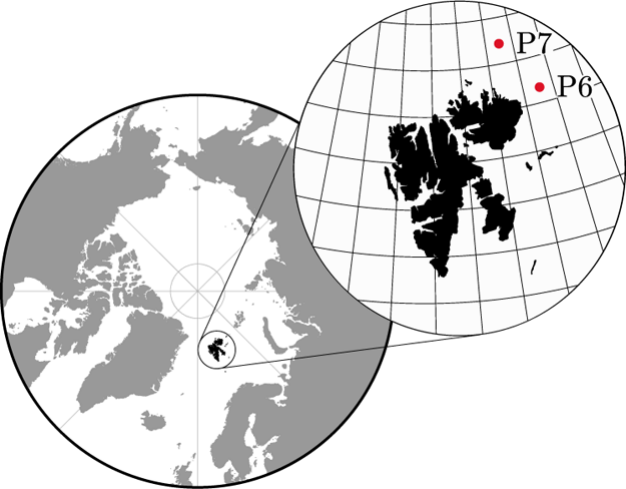
Locations of sampling stations P6 (81°16′N, 31°75′E) and P7 (81°16′N, 29°75′E) during Nansen Legacy Q3 summer cruise in August 2019.

Environmental samples were collected in the European High Arctic (see Figure 1) during the “Nansen Legacy Q3” summer cruise of the Norwegian research vessel *Kronprins Haakon* on 26–28 August 2019. Samples were taken at two study sites situated on the Barents Sea shelf break (81°16′N, 31°75′E) and Nansen Basin (81°16′N, 29°75′E), which we refer hereafter as P6 and P7, respectively. Both sites were selected on separate large (≈1 km^2^) undeformed ice floes that were fully covered with a thin layer of snow (≈0.05 m) except in areas where melt ponds were present. Seawater samples (1 L; *n* = 6) were collected at two depths (0.5 and 5.0 m) using a Niskin bottle via an access hole in the sea ice. Sea ice and snow were collected in close proximity (<20 m) to the seawater access hole.

Melt ponds were covered by a thin layer of ice, which was removed before sampling water (1 L; *n* = 3). Snow samples (2 L; *n* = 4) were collected above each core site (approximately 0.25 m^2^). Sea ice cores (*n* = 4) were drilled (spaced ≈1 m intervals) and then immediately cut into horizontal sections (*n* = 7–10) that were between 0.1 and 0.2 m in length. Ice core sections were bulked with adjacent ice core samples (see Figure S1) to obtain sufficient meltwater for PFAS analysis (≈1 L) and placed into polyethylene bags before melting at ambient room temperature (≈20 °C). All equipment and sampling bottles (polypropylene) for PFAS analysis were pre-cleaned following set protocols, and any clothing worn by samplers that was suspected to contain residues of fluoropolymers was avoided to prevent possible contamination. For more information on sampling protocols, equipment, and ancillary measurements (e.g., bulk density and temperature) see Tables S1–S5 and Figure S2.

### 2.2 Sample Extraction and Analysis

Solid-phase extraction (SPE) of PFASs took place onboard the ship, and several procedural blanks (*n* = 7) were taken to assess possible contamination. Briefly, an internal standard (IS) mix (^13^C mass-labelled standards) was added to each sample before being loaded onto pre-conditioned OASIS WAX cartridges (150 mg, 30 μm, 6 mL). Cartridges were then dried under vacuum and stored at −20 °C before further analysis at Helmholtz-Zentrum Hereon, Germany. Instrumental analysis of PFASs was performed by high-performance liquid chromatography tandem mass spectrometry, using an HP 1100 LC system (Agilent Technologies, USA) coupled to an API 4000 triple quadrupole mass spectrometer (AB Sciex, USA). It was equipped with a Turbo V ion source (AB Sciex, USA), operating in the negative electrospray ionization mode. For chromatographic separation, a polar-embedded reversed-phase C_18_ separation column (Synergi Fusion-RP C_18_, Phenomenex, USA) was combined with a reversed-phase guard column (Phenomenex, USA). As solvents for the gradient elution, 2 mM ammonium acetate aqueous solution (A) and 0.05% acetic acid in methanol (B) were used. The injection volume was 10 μL for samples and standards, both dissolved in 80:20 (% v/v) methanol/water. Target compounds included 11 PFCAs (C_4_–C_14_) and 5 PFSAs (C_4_, C_6_–C_8_, and C_10_). See Table S6 for more information on analytical standards.

Aliquots of each sample (0.05 L) were also taken for salinity and stable oxygen isotope analysis that were stored at 4 °C in airtight bottles with minimal headspace for <14 days before analysis was undertaken at Lancaster University, UK. Salinity was measured (all volume concentrations reported for 20 °C) using a calibrated conductivity probe (Hach HQd40 logger with the CDC401 probe) and reported as parts per thousand (ppt or g/L). Sample ^18^O/^16^O ratios in water were determined by continuous-flow isotope-ratio mass spectrometry at the University of Lancaster (Elementar pyrocube elemental analyzer linked to an Isoprime 100 mass spectrometer). Sample δ^18^O analyses were undertaken in the pyrolysis mode using sample injection of 0.4 μL over glassy carbon chips at 1450 °C. δ^18^O values were corrected against laboratory calibration standards (per mille, ‰) relative to Vienna Standard Mean Ocean Water (V-SMOW; δ^18^O = 0 ‰) and Greenland Ice Sheet Precipitation (GISP; δ^18^O_H2O_ = −24.78‰). Laboratory standards containing NaCl in similar proportion to the field samples were also used to check for any effects of salinity on the true sample value. Results confirmed no adverse effect of salinity on analytical precision or accuracy. Within-run standard replication for δ^18^O was <0.2‰ (1.s.d.).

### 2.3 Quality Assurances and Data Analysis

All reported PFAA concentrations were recovery-corrected using a mass-labelled analogue unless stated otherwise (see Table S7). Analytical recovery (%) and analytical precision (%) are displayed in Figures S3 and S4, respectively. Method detection limits were determined for each PFAA (MDL: *x*_procedural blank_ + 3·s.d_procedural blank_) using procedural blanks (*n* = 7) that comprised laboratory (*n* = 5; SPE-filtered milliQ water) and field blanks (*n* = 2; collected washings of field sampling equipment with SPE-filtered milliQ water). Concentrations of PFAA in samples that were below method detection limits were considered as non-detects, and sample values were then subject to blank subtraction using an average of all procedural blanks (*n* = 7). All non-detects were taken as zero, and all blank-corrected values (see Table S8–S12) were included in further calculations (see eqs S1–S13) and statistical testing. Statistical analyses were performed using concentration data (pg L^–1^) in RStudio (version 1.1.453; RStudio Team, 2015) using a significance level of α = 0.05. Normality was tested using the Shapiro–Wilks test, before further statistical comparisons. Significant differences in PFAA concentrations were determined using Wilcoxin’s signed-rank test. Spearman’s correlation analysis was used to investigate relationships between PFAA and other physical properties in sea ice. To fully evaluate the origin of water in sea ice and assess the contribution of meltwater to the surrounding sea ice system, volumetrically weighted concentrations were calculated (see eqs S4–S7).

## 3 Results and Discussion

### 3.1 Sea Ice Origin and Characteristics

**Figure 2:**
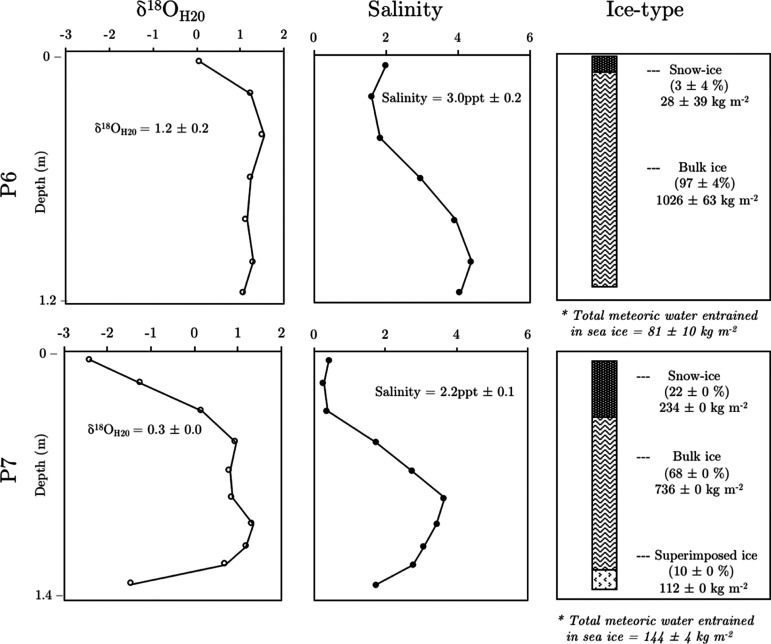
Physical sea ice properties and mass of each ice-type at P6 and P7. The ice-type is determined using δ^18^O_H20_ values, which varies depending on the amount of entrained meteoric water. Snow-ice = δ^18^O_sea ice_ values < 1‰; bulk ice = δ^18^O_sea ice_ values 1–3‰; and superimposed ice = δ^18^O_sea ice_ values < 1‰ (salinity <2 ppt). Snow is not shown in this figure but possessed δ^18^O_H20_ values < 10‰. The amount of different ice-types (%) is calculated as a fraction of the total mass of water (kg m^–2^) in the sea ice column. Points represent ice core samples approximately 0.1–0.2 m in length. *The total amount of meteoric water based on isotopic fractionation in newly formed sea ice of δ^18^O_newly formed sea ice_ = 2.6‰.^22^

Sea ice is a heterogeneous matrix of ice formed from seawater, however, which over time may have also been influenced by atmospheric inputs (meteoric) through precipitation such as snow. Figure 2 illustrates some of the physical characteristics of sea ice collected at sampling stations P6 (top panels) and P7 (bottom panels). Mean salinities (volumetrically weighted) in sea ice (<3.0 ppt) at both P6 and P7 were significantly lower than those of seawater (32 ppt) but higher than those of snow (<0.1 ppt). As sea ice forms and grows during winter, entrapped seawater is concentrated (due to the freezing-out effect) and is rejected from the sea ice matrix as brine (a super-saline solution present within the bulk sea ice) into the underlying ocean through a process commonly referred to as gravity drainage.^18^ A small amount of salt does, however, become incorporated into the bulk ice.^19,20^ Low salinities are therefore typical in first-year sea ice and multiyear ice (MYI), particularly during late summer when additional ice mass may also have been gained through the incorporation of fresh snow. Furthermore, sea ice may have undergone surface “flushing” of fresh meltwater, a process that occurs frequently during the Arctic summer^21^ and also serves to remove salts from sea ice. While the sea ice was thinner at P6 (1.2 ± 0.1 m) compared to that in P7 (1.4 ± 0.1 m), both showed a vertical “S”-shape salinity profile, where layers at the snow–ice interface were significantly fresher, indicating the influence of snow water on the distribution and likely dilution of salt in the upper ice layers (Figure 2).

The influence of snow on sea ice properties was also supported by measurements of δ^18^O_H20_ values, which revealed δ^18^O_H20_ values in sea ice (δ^18^O_sea ice_ = 0.5 ± 1.2‰) that were significantly more positive than those in pure snow (δ^18^O_snow_ = −15.2 ± 1.1‰) and seawater (δ^18^O_seawater_ = −0.1 ± 0.4‰). During freezing of ocean water to form sea ice, equilibrium fractionation of oxygen isotopes in water enriches δ^18^O_sea ice_ by approximately 2.6‰.^22^ This generates isotopic signatures in sea ice that are enriched in ^18^O compared to precursor seawater values. The measurement of δ^18^O_sea ice_ values which are frequently isotopically lighter than seawater values indicates the entrainment of meteoric water, which is expected to have occurred during the aging of sea ice at both sites. One potential other source of meteoric water to sea ice is from glacial run-off entering into fjord environments/coastal fringes of the Barents Sea^23^ during the summer season. However, given the sampling locations in this study and the volume of glacial meltwater required to lower the isotopic composition in sea ice by the required amount, the incorporation of atmospheric snowfall is the most likely source of meteoric water.

Similar to salinity profiles, δ^18^O_H2O_ values at the surface of sea ice at both P6 and P7 were lower compared to those in deeper ice layers, which indicates a strong influence of atmospheric precipitation (probably mostly snow) on the composition of sea ice at both sites. However, a comparison of δ^18^O_sea ice_ profiles at P6 and P7 also revealed some marked differences between sea ice at either sites. A notable feature at P7 was low δ^18^O_sea ice_ values (δ^18^O_sea ice_ < −2‰) in the deepest ice situated at the seawater–ice interface. One explanation for this observation is the percolation and subsequent refreezing of meltwater derived from surface precipitation, forming an ice layer known as superimposed ice. This is also supported by salinity measurements, which show that the bottom layers of sea ice at P7 were notably fresher. Superimposed ice forms when the surrounding ice matrix is colder than the freezing temperature of freshwater^24^ and is a common feature in Arctic and Antarctic glaciers. Superimposed ice has also been reported in sea ice,^25,26^ and its presence suggests that the ice at P7 was second-year ice (SYI) or MYI.

In order to differentiate between compartments of sea ice that were influenced by differing quantities of water of meteoric origin, sea ice was classified into three broad ice-types based on δ^18^O_H20_ values (and salinity): (i) “Snow-ice” (δ^18^O_sea ice_ values < 1‰) that retains a strong meteoric signal; (ii) “bulk ice” (δ^18^O_sea__ice_ values 1–3‰) predominantly influenced by sea water and less by snow meltwater; and (iii) “superimposed ice” (δ^18^O_sea ice_ values < 1‰ and salinity <2 ppt), present at deeper layers and influenced by snow meltwater (or other high-latitude atmospherically derived precipitation). Using the cumulative ice sample length (m) and measured ice densities (kg m^–3^), the mass or load of water (kg m^–2^ ice column) was calculated for each of the respective ice-types (see Figure 2; right panel). Results showed that while snow-ice was present at both sites, the relative amount (% snow-ice mass/total sea ice mass) varied substantially between P6 (7 ± 2%) and P7 (22 ± 0%). Snow-ice is formed when free floating sea ice receives a substantial deposition of snow, which in turn leads to seawater flooding of the surface sea ice and basal snow layers, forming a salty slush layer that can then refreeze.^27^ Snow-ice is therefore a mixture of snow and seawater.^28^ Such a large difference between the ice floes indicates that the two sites comprised sea ice of different ages and/or had followed different “lifecycles”. Increased snow-ice at P7 indicates that the ice floe had retained a greater amount of its snow loading from the previous winter season. Established methods (Granskog et al., 2017)^21^ were used to investigate this further by calculating the relative amount (% meteoric water mass/newly formed sea ice mass) of meteoric water incorporated within the ice floes at P6 and P7, based on an initial isotopic fractionation in newly formed sea ice of δ^18^O_newly formed sea ice_ = 2.6‰.^22^ This indicated that sea ice at P6 and P7 was composed of 8 ± 1 and 13 ± 0 % meteoric water, respectively. These estimations of entrained meteoric water in the respective ice floes correspond well with those given by Granskog et al., (2017) for first-year sea ice (FYI; 3.3–4.4%) and SYI (12.7–16.3%) sampled in a comparable region (adjacent to the Barents Sea) during winter. This suggests that sea ice at P6 and P7 is FYI and SYI (or older), respectively, and supports the assertion that P7 had received a higher snow load (probably over successive seasons) compared to P6. While our values of meteoric water content for P6 ice are slightly higher than those reported by Granskog et al., our samples of sea ice were collected during late summer and hence may have entrained more precipitation (i.e., snow or rain) or been further influenced by freeze–thaw cycles and/or drainage of surface snow-derived meltwater during summer.

### 3.2 Occurrence of PFAA in Snow, Sea Ice, and Seawater

Table 1 shows a summary of measured concentrations of PFAAs in different environmental compartments sampled during the cruise (the complete data set can be found in the Supporting Information). Notable differences in concentrations (and environmental behaviors) were observed between the short- and long-chain PFAAs, and therefore, results for each group are reported accordingly. In snow samples, most target PFAAs were detected, with the exception of PFBS (C_4_) and PFOS (C_8_) and showed similar levels across both sites (*n =* 6). Consequently, concentrations in snow (meltwater equiv.) were averaged, and values hereafter refer to the mean concentrations from both sites. The presence of PFAAs in the snowpack implies a meteoric source, signifying atmospheric transport as a major pathway to the Arctic marine environment, although a fraction of PFAAs in ice-rafted snow may also have arisen from marine aerosol generated from sea spray.^29^ Concentrations of short-chain PFAAs in snow in this study were significantly higher (Wilcoxon signed-rank test, *p* < 0.01) than those of long-chain PFAAs. Similar findings were reported in snow (see Table 1) sampled from sites located in the Western Arctic^5^ and others^6,7^ in the Canadian Arctic Archipelago, suggesting that similar chemical sources influence different Arctic regions. Nevertheless, concentrations of short-chain PFAAs in snow were generally much higher in our study driven notably by high levels of perfluorobutanoic acid (PFBA = 2.6 ± 0.7 ng L^–1^), which probably reflects the higher industrial consumption of chemical precursors resulting in its formation in recent years. Concentrations of long-chain PFAAs in snow, however, were similar to those measured in previous studies,^5,7^ which shows that atmospheric deposition, presumably through the photo-oxidation of volatile precursors (e.g., fluorotelomer alcohols), is still ongoing at the same rate over the last 10 years or so.

**Table 1:** Summary of the Measured Concentrations of Short- and Long-Chain PFAAs in Relevant Compartments Reported in Key Studies throughout the Arctic

chemical	ΣPFAA_short-chain_ (ng L^–1^)				ΣPFAA_long-chain_ (ng L^–1^)			
snow	0.7 ± 0.6	n/a	n/a	2.9 ± 0.8	0.2 ± 0.1	n/a	n/a	0.2 ± 0.1
sea ice	0.3 ± 0.5	n/a	n/a	1.9 ± 2.8	0.6 ± 0.4	n/a	n/a	0.2 ± 0.2
seawater	0.3 ± 0.2	0.1 ± 0.0	0.3 ± 0.0	0.6 ± 0.1 (0.5 m)	0.1 ± 0.0	0.1 ± 0.0	0.4 ± 0.0	1.4 ± 0.9 (0.5 m)
				0.2 ± 0.1 (5.0 m)				0.4 ± 0.2 (5.0 m)
location	Western Arctic Ocean (ice-free)	Greenland Sea (ice-free)	North Sea (ice-free)	Barents Sea region	Western Arctic Ocean (ice-free)	Greenland Sea (ice-free)	North Sea (ice-free)	Barents Sea region
reference	Cai et al., 2012	Joerss et al., 2020	Joerss et al., 2020	this study	Cai et al., 2012	Joerss et al., 2020	Joerss et al., 2020	this study

Average concentrations (mean ± s.d.) use all data from P6 and P7 sampling stations (∼81 °N). Surface seawater in the North Sea (∼58–62 °N) and Greenland Sea (∼68–79 °N) was taken along a latitudinal transect and sampled at a depth of 11 m. Surface seawater (∼66–70 °N) and sea ice (∼77–87 °N) samples analyzed by Cai et al. (2012) were collected in different areas of the Western Arctic Ocean, and the seawater sampling depth was not explicitly stated. Concentrations listed in sea ice in this study are volumetrically weighted. n/a = no available data. Note: ΣPFAA concentrations listed in this study may differ slightly from those stated in the original reference due to the selection of PFAAs to match the same target PFAAs as in this study.

**Figure 3:**
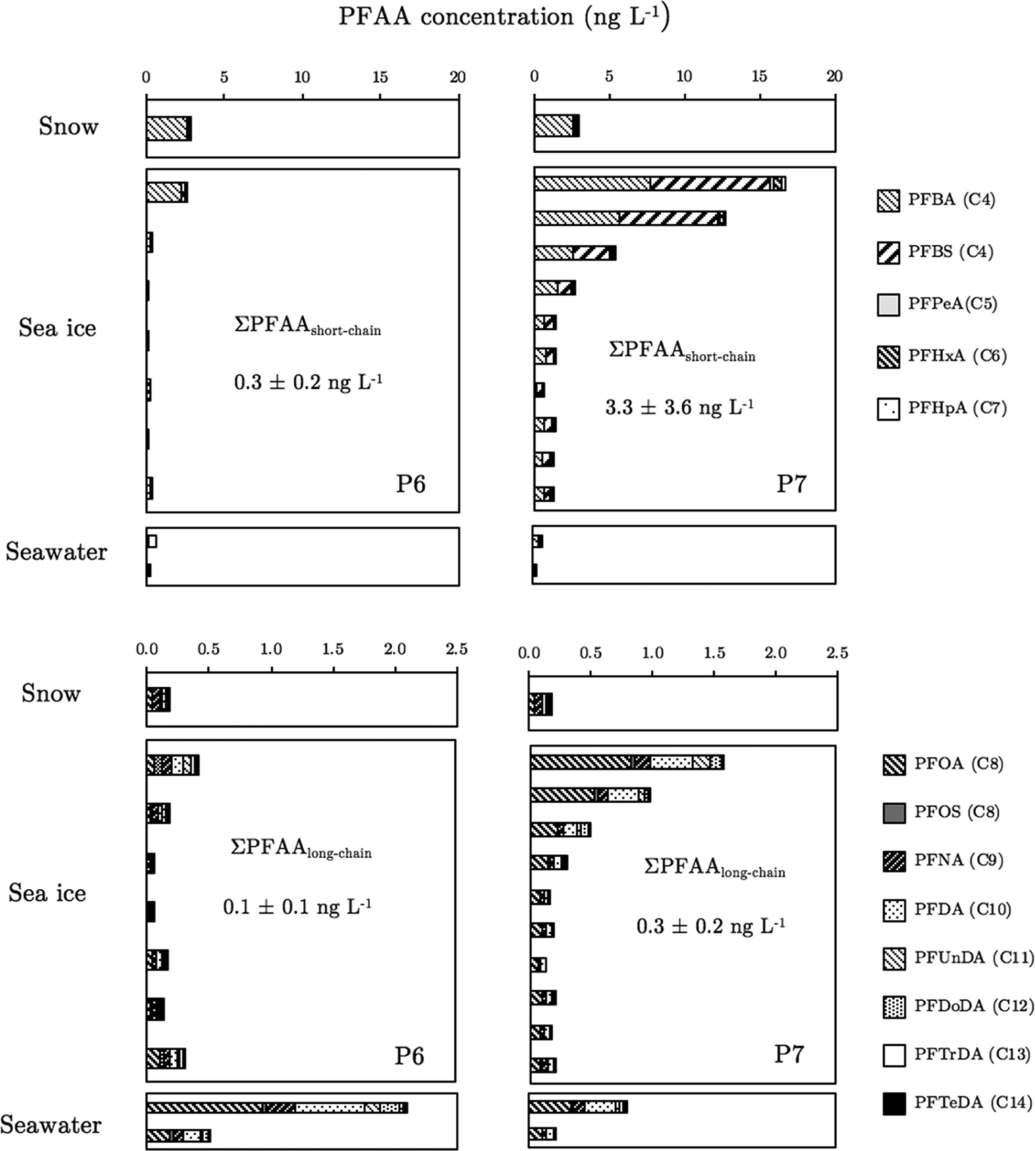
Sum of short- and long-chain PFAAs in different environmental compartments. Concentration profiles for short- and long-chain PFAAs are shown in the upper and lower panels, respectively. Concentration profiles for P6 and P7 are shown in the left and right panels, respectively. Seawater samples collected at a depth of 0.5 and 5 m are also represented by the upper and lower bar, respectively. Low concentrations of short-chain PFAAs (e.g., PFBA) in seawater may be attributed to analytical issues related to matrix effects. The sum (mean ± s.d.) of short- and long-chain PFAAs in sea ice (volumetrically weighted) is also included in each panel.

PFCAs and PFSAs are formed in the atmosphere from a wide number of precursor chemicals, and positive correlations between the various PFAAs suggest that they share similar sources and/or transport pathways into the Arctic (see Figure S4). While the presence of relatively high levels of PFBA (C_4_) is probably linked to chlorofluorocarbon-replacement compounds,^6^ other important precursors include perfluoroalkane sulfonyl fluorides and fluorotelomer-based compounds (FT-based).^30^ The presence of the long-chain PFCAs such as PFDA (C_10_) and PFUnA (C_11_) (not primary substances in commercial products) is most likely through the atmospheric photochemical transformation of fluorotelomer chemicals including olefins,^31^ acrylates,^32^ and iodides^33^ and alcohols.^34,35^ Interestingly, PFBS (C_4_) and PFOS (C_8_) were the only PFSAs detected in this study, which shows they are still the main perfluoroalkyl sulfonates present in the Arctic environment,^36^ but they were below detection limits in our snow samples. While low levels of PFOS in snow may be related to industry initiatives aimed to lower environmental emissions of PFOS and related compounds (i.e., C_8_-based), remote Arctic snow is still expected to contain levels of PFBS (C_4_) due to the increasing use of analogous short-chain products (i.e., C_4_-based) as replacements to sustain global demand of high performing surface active agents.^37^ This shows that our current understanding of the sources and environmental processes that govern the transport and cycling of some PFAAs in the Arctic environment is incomplete and warrants further research. Nevertheless, given that PFOS have recently been detected in most snow samples from the Canadian Arctic,^7^ the absence of PFSAs in our snow samples shows that concentrations of some compounds vary considerably. This could be related to to seasonal changes in photochemical activity^38^ or even seasonal variations in the atmospheric transport of specific volatile precursor compounds to the Arctic.^39^ However, it is also likely that PFOS and PFBS (and probably other PFAAs) have undergone early elution from the snowpack in meltwater during snow ageing/thawing episodes,^40−42^ as indicated by high levels of PFBS in sea ice layers directly beneath the snow (see Figure 3).

The mean concentration of both short- and long-chain PFAAs in sea ice was comparable to that of snow, with short-chain PFAAs also showing similarly higher levels than long-chain PFAAs (Wilcoxon signed-rank test, *p* < 0.05). Figure 3 illustrates the vertical concentration profiles of short- (top panels) and long-chain (bottom panels) PFAAs in sea ice at P6 (left panels) and P7 (right panels). Concentrations in the surface layers of sea ice tended to be much higher than in lower layers. The higher concentrations of PFAAs in the surface sea ice layers correspond well with patterns observed in sea ice located in other regions of the Arctic,^5^ indicating similar Arctic-wide processes affecting PFAA accumulation in sea ice. Elevated concentrations of PFAAs in the uppermost ice layers of sea ice may be a result of entrainment of chemicals present in seawater during initial sea ice formation.^15,43^ However, snow is also likely to play an important role in the delivery of PFAAs^44^ to sea ice with the amount and timing of snowfall deposition likely to affect accumulation dynamics.

Differences were apparent between the concentrations and distribution of PFAAs in sea ice at both sites, notably in the general shape of the concentration profiles. A gradual reduction from high concentrations at the surface toward lower ice layers was seen for short-chain PFAAs at P7, but a more marked reduction was observed at P6. Indeed, many short-chain PFAAs were below method detection limits in the deeper ice layers (>70% of sea ice samples) at P6, and this demonstrates the different ice ages and “weathering” processes (i.e., freeze–thaw activity) between these two sites. The short-chain PFAAs are more water-soluble than the long-chain PFAAs and likely to be more mobile when liquid water is present in the ice system.^41−43^ Preferential elution of short-chain PFAAs down a snow core has been observed in a temperate Tibetan mountain glacier that experienced summer melt episodes,^45^ and hence, the absence of the short-chain PFAAs in the deeper sea ice at P6 most likely indicates loss through elution by meltwater drainage with subsequent replenishment in the surface layers through snow deposition. At P7, this is not apparent, and the higher concentrations of short-chain PFAAs down the ice core show that meltwater drainage was more limited compared to P6. While the presence of superimposed ice at the bottom of the sea ice at P7 (see Figure 2; right panel) may affect the downward percolation of PFAA in meltwater,^46^ it is more likely that less thawing occurred at P7, which in turn preserved the accumulated burden of PFAAs. The exact reason for this difference in “weathering” history (i.e., melt rate) between sites is unknown but could be due to greater inflow of warmer Atlantic water at P6 compared to P7^47^ and/or related to the amount of snowfall (see Section 3.3) serving as insulation against atmospheric thermal changes.^48^

Long-chain PFAAs also showed a gradual decrease in concentrations from the surface ice layers of sea ice but with an increase in concentrations in lower layers (notably at P6) to form a concentration profile that resembled a “C” shape. A c-shape concentration profile in sea ice is typical for salt (NaCl) and other dissolved solutes such as PFASs^43^ and even hydrophobic organic pollutants^15^ during winter. This feature develops through a process known as gravity drainage, whereby chemical constituents present in freezing seawater are excluded from the pure ice crystal matrix and rejected into adjacent brine channels.^20^ The stronger resemblance of this c-shape concentration profile for long-chain PFAAs at P6 compared to P7 therefore supports the previous assertion that sea ice at P6 and P7 comprises FYI and SYI (or older), respectively, as discussed in Section 3.1. Furthermore, the distinct concentration profiles between the short- and long-chain PFAAs illustrate the differences in their physical–chemical properties and their subsequent environmental behavior during the transition toward the summer ice system.

Unlike snow and sea ice which showed higher concentrations of short-chain PFAAs, the under-ice seawater revealed greater concentrations of long-chain PFAAs compared to short-chain PFAAs (see Table 1). This is likely to be a reflection of the longer-term input of PFOA and other long-chain PFAAs to the Arctic seawater over the last few decades.^7,49,50^ Concentrations of PFAAs in the seawater directly below the ice (0.5 m depth) also support this by revealing significantly higher (Wilcoxon signed-rank test; *p* < 0.05) values than at the greater depth of 5 m at both sites (see Figure 3). A comparison of mean total PFAA concentrations (i.e., short- and long-chain PFAAs; ΣPFAA) in seawater samples at 5 m in this study (ΣPFAA = ≈0.6 ng L^–1^) with recent measurements made in ice-free parts of the Greenland Sea (ΣPFAA = ≈0.4 ng L^–1^) showed that our concentrations are comparable to previous observations. In contrast, seawater concentrations at 0.5 m in this study (ΣPFAA = ≈2.0 ng L^–1^) were ≈5-fold more and were even higher than those measured in surface seawater (ΣPFAA = ≈0.7 ng L^–1^)^4^ in the North Sea affected by industrial inputs of PFAAs. Given the remoteness of the sampling sites in this study, the lack of local sources,^51^ and low blank values (that rule out potential contamination artifacts), the high concentrations of PFAAs in seawater in close proximity to sea ice must be driven by the overlying ice pack through release from meltwater drainage given the time of year when sampling occurred. This is supported by meteorological data (Tables S4 and S5) and measurements made on the sea ice cores (Figure S2) that show that temperatures were high enough to cause some melting. Differences in concentrations between the two depths (0.5 and 5 m) are unlikely to be caused by sampling different stratified water masses, as the polar mixed layer extends from the surface to ∼10 m depth in the Barents Sea under summer ice-covered conditions^52^ and is characterized by slightly lower salinity compared to deeper waters. These findings are analogous to a fresh water column in a lake located in the Canadian High Arctic, whereby elevated concentrations of PFAAs were found in surface waters following the onset of melt.^53^ Although higher concentrations of short-chain PFAAs in under-ice seawater were not as marked relative as those of long-chain PFAAs, this probably reflects the earlier elution of these particular chemicals from the ice pack with subsequent dispersal. It is also noteworthy that the analytical recovery of some short-chain PFAAs in seawater samples was low (e.g., PFBA <10%; see Figure S3, with an analytical precision of 50% RSD for seawater; see Figure S4). Given that PFBA contributed >90% of the sum of short-chain PFAAs in snow/sea ice samples (Section 3.2), it is therefore likely that the concentration of PFBA is bias low, and thus, the concentration of short-chain PFAAs in seawater is underestimated. Nonetheless, taking this possible artifact into consideration, PFAA concentrations in under-ice seawater in this study were still higher than those observed in ice-free zones in the North Sea by Joerss et al. (2020),^4^ who used the same analytical methodology.

### 3.3 Thawing Ice Pack Influences PFAAs in the Under-Ice Seawater

**Figure 4:**
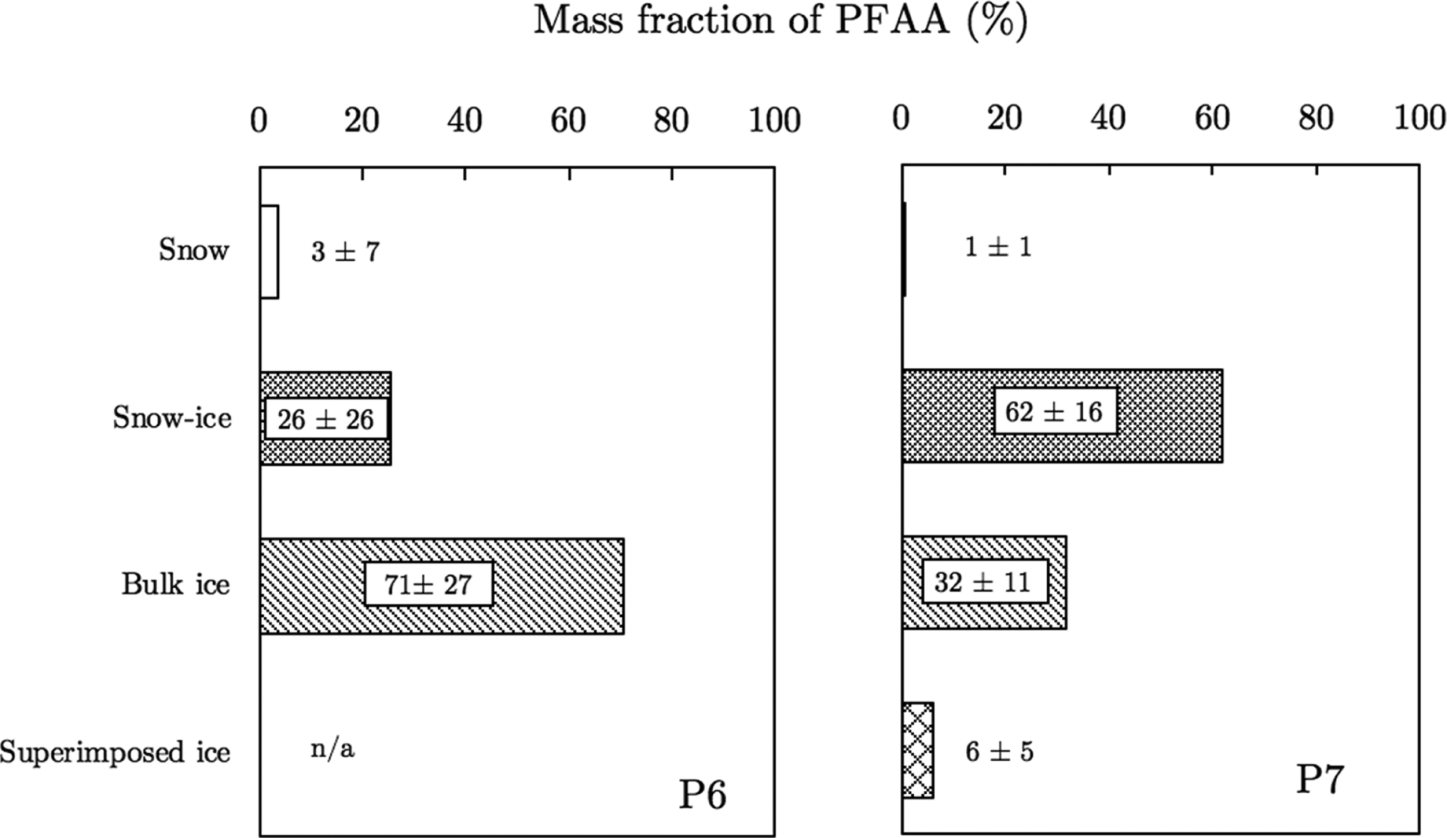
Mass apportionment of ΣPFAA (i.e., short- and long-chain PFAAs) in different “ice-types” at the sampling stations, P6 and P7. n/a = not applicable.

The concentrations of PFAAs measured in the under-ice seawater (0.5 m) at P6 were the highest in this study (e.g., ΣPFAA_P6_seawater_0.5 m_ = 2.7 ± 1.5 ng L^–1^), which suggests that PFAAs originate from the overlying melting ice pack. Although the PFAA profile (% ΣPFAA_long-chain_) in seawater was not significantly different between sites (see Figure S6), concentration ratios for individual PFAAs (e.g., *c*(PFAA)_P6_seawater_0.5 m_/*c*(PFAA)_P7_seawater_0.5 m_) revealed that PFAA concentrations at P6 were on average 4-fold higher than at P7, and this is likely to be related to the different thawing or “weathering” history of the overlying ice pack at the two study sites. To investigate this further using a mass-apportionment approach, Figure 4 shows the mass fraction of all PFAAs (% ΣPFAA) in the different ice-types at P6 (left panel) and P7 (right panel). While large proportions of PFAAs were contained within bulk ice (32–71% ΣPFAA), which made up the majority of the total water mass in the ice systems across both sites (see Figure 2), high proportions of PFAAs were also present in snow-ice (26–62% ΣPFAA). This ice-type, however, made a relatively minor contribution to the total water mass of the ice pack at both sites (3–22%). Similarly, the ice-rafted snow layers contained 1–3% ΣPFAA burden at both sites but comprised <1% of the total water mass of the respective ice systems. Substantial loss of surface layers, including snow and snow-ice, during periods of thaw will therefore mobilize portions of the sea ice pack that contain a high burden of PFAAs (see Tables S13–S14), which in turn will lead to significant increases in PFAA concentrations in the receiving under-ice seawater, particularly when atmospheric temperatures rise through seasonal changes.

Using an average (mean ± s.d.) snow depth of 0.49 ± 0.13 m and snow density of 363 ± 24 kg m^–3^ for the snowpack across a comparable region (north of Svalbard) during winter^21^ gives rise to an estimated annual snow water equivalent load of 178 ± 49 kg m^–2^. Mass-apportionment calculations utilizing mean δ^18^O_snow_ values (assuming constant sea ice mass) show only 81 ± 10 kg m^–2^ of entrained meteoric water at P6, whereas the entrained meteoric water mass at P7 is higher at 144 ± 4 kg m^–2^ and closer to the annual snow water equivalent value mentioned above. This demonstrates that a substantial loss of the annual snowfall occurred over the course of the season at P6, resulting in the lower overall mass of the entrained meteoric water. The loss of meteoric water at P6 is likely to have occurred through thawing of snow and snow-ice layers followed by meltwater percolation through the ice into the under-ice seawater. In turn, this meteorically influenced meltwater will transfer its high PFAA burden either deeper into the bulk ice or into the under-ice seawater. In contrast, the higher mass of meteoric water at P7, an ice floe likely comprising older ice, demonstrates relatively less thawing during its recent history and hence lower release of meteorically derived meltwater compared to P6. As a consequence, high concentrations of PFAAs in sea ice are retained in snow-ice and other upper sea ice layers, enabling chemicals to accumulate and concentrations to exceed those in sea ice at P6.

The composition (% ΣPFAA_long-chain_) of sea ice and adjacent compartments also indicates that the ice floes at both sites had been influenced by distinct processes, leading to different PFAA patterns (Figures S6 and S7), with P6 and P7 being only weakly correlated (*r*^2^ = 0.30, *n* = 8; *p* > 0.15) with each other. Sea ice composition at P6 was more highly correlated with snow (*r*^2^ = 0.77, *n* = 8; *p* < 0.01) than seawater, which implies that the ice floe was affected by recent meltwater arising from thawing of moderately fresh snow. Thus, the lower entrainment of meteoric water at P6 via snowfall, as indicated by isotopic measurements, illustrates that melting of surface sea ice layers (i.e., snow and snow-ice) probably occurred earlier in the season with later melt originating from fresh snow. Conversely, sea ice at P7 was more highly correlated with seawater (*r*^2^ = 0.98, *n* = 8; *p* < 0.001) than snow, which suggests less thawing and greater surface flooding of sea ice with seawater (i.e. snow-ice). The excellent agreement between stable isotope measurements and PFAA observations made on the same samples in this study provides high confidence that our account of the evolution history of sea ice at both sites is accurate.

### 3.4 Melt Pond Significance and Environmental Implications

This study provides compelling evidence that the melting of the marine ice pack drives high concentrations of PFAAs in under-ice seawater and serves as a significant source of these chemicals to the under-ice environment during seasonal thaw. Although PFAAs in sea ice may also originate from the uptake of PFAAs from seawater during its initial formation, the data presented in this study show that a high proportion of PFAAs is present in snow-ice and hence probably derived from the atmosphere. This demonstrates that snow plays an important role in both the delivery and storage of PFAAs in the sea ice pack, with the timing and amount of snowfall likely to influence the melting and subsequent release of PFAAs into the seawater.

We also measured PFAAs in water samples collected from melt ponds that were present on the ice floes at P6 and P7. Melt ponds feature on the surface of predominantly late-season sea ice in the Arctic and form as snow and sea ice thaw.^54^ At more advanced stages, melt ponds are influenced by intrusions of seawater and they have been shown to play an important ice-mediated annual delivery role of some semi-volatile contaminants (e.g., organochlorine pesticides) to the under-ice seawater.^17^ Given the very different physico–chemical properties of PFAAs compared with these other previously studied organic contaminants, investigating the significance of melt ponds on their environmental cycling is warranted. By comparing the melt pond composition (e.g., salinity and δ^18^O_H2O_) with the surrounding endmembers (i.e., snow, sea ice, and seawater), their meteoric/oceanic origin can be established. Using average values of salinity and δ^18^O_H2O_ in endmembers (Table S15), the mass fraction (%) that each of these three sources contributed to individual melt ponds can be calculated.^55^ The results shown in Table S16 suggest that snow (35 ± 9%) and sea ice (64 ± 10%) were the major contributors to the composition of melt ponds, with only a small fraction coming from seawater (0 ± 2%). The mass fraction (%) and measured PFAA concentration (ng L^–1^) in each respective endmember were multiplied and then summed to give “predicted” concentrations of each PFAA in melt pond water (Tables S17 and S18). In general, the measured concentrations of PFAAs in melt ponds were considerably lower than in the under-ice seawater, and a comparison between the “measured” and “predicted” PFAA concentrations in melt ponds revealed no significant difference (Wilcoxon signed-rank test, *p* < 0.05). This shows that the melt ponds we sampled were not a significant source of PFAAs and are unlikely to have played a role in the high PFAA concentrations measured in seawater. These results support the assertion that the melt ponds had formed during the later stages of the Arctic summer season after significant thawing had already occurred. Together, this information strongly suggests that the occurrence of high PFAA concentrations in under-ice seawater is attributed to thawing of surface sea ice layers early on in the season and shows that different contaminant classes (e.g., organochlorine pesticides, PFAAs, etc.) do not necessarily follow the same environmental cycling patterns in the marine polar environment.

The concentrations of short- and long-chain PFAAs in under-ice seawater are comparable to levels observed in coastal seas in temperate latitudes, but the duration of these high concentrations is probably dependent on the presence of different ice-types. Following complete ice breakup and thawing, concentrations in surface seawater are likely to decline. Nonetheless, the presence of PFAAs in the under-ice environment at concentrations comparable to those of temperate coastal seas which are influenced by direct inputs of pollution presents an exposure hazard to ice-associated biota and organisms at the base of the pelagic food web. Further efforts are now required to investigate the duration and timing of these periods of elevated concentrations and how they link with biological events such as planktonic blooms. Although more work is needed to ascertain the impact of high concentrations of PFAAs on biological communities, the work presented in this study shows that the thawing of the ice pack may present an efficient pathway for these chemicals to enter the base of the Arctic marine food web.

## Supporting Information Available

List of target analytes and quality assurance criteria and concentration, meteorological, density, and temperature data with all associated equations (PDF)
