# Supplementary material for: High Concentrations of Perfluoroalkyl Acids in Arctic Seawater Driven by Early Thawing Sea Ice
Source: Environ Sci Technol. 2021 Jul 26;55(16):11049–59. doi: 10.1021/acs.est.1c01676 (PMC8383270; doi:10.1021/acs.est.1c01676)
Supplement: Supplementary file 1 — es1c01676_si_001.pdf [file es1c01676_si_001.pdf]

Supporting Information to:

High concentrations of perfluoroalkyl acids (PFAA) in Arctic seawater driven by early thawing sea ice.

Jack Garnett[1], Crispin Halsall[1*], Anna Vader[2],
Hanna Joerss[3], Ralf Ebinghaus[3], Amber Leeson[1], Peter M. Wynn[1]

1 Lancaster Environment Centre, Lancaster University, Lancaster, LA1 4YQ, UK
2 Department of Arctic Biology, The University Centre in Svalbard (UNIS), N-9170 Longyearbyen, Norway
3 Helmholtz-Zentrum Hereon, Max-Planck-Straße 1, 21502 Geesthacht, Germany

Email: c.halsall@lancaster.c.uk

Contents include:

21 pages
Tables (S1-S18)
Figures (S1-S7)
Equations (S1- S13)

Table S1: Sea ice sampling depths at P6 and P7

Site	Sample section length (m) (n=4)	Mean cumulative depth (m) (n=4)
P6	0.10 ± 0.00	0.00 – 0.10
	0.20 ± 0.00	0.10 – 0.30
	0.20 ± 0.00	0.30 – 0.50
	0.20 ± 0.00	0.50 – 0.70
	0.20 ± 0.00	0.70 – 0.90
	0.20 ± 0.00	0.90 – 1.10
	0.10 ± 0.00	1.10 – 1.20
		Total = 1.20 ± 0.0
Site	Sample section length (m) (n=4)	Mean cumulative depth (m) (n=4)
P7	0.10 ± 0.00	0.00 – 0.10
	0.15 ± 0.00	0.10 – 0.25
	0.18 ± 0.03	0.25 – 0.43
	0.18 ± 0.05	0.43 – 0.60
	0.15 ± 0.00	0.60 – 0.75
	0.15 ± 0.00	0.75 – 0.90
	0.15 ± 0.00	0.90 – 1.05
	0.10 ± 0.00	1.05 – 1.15
	0.10 ± 0.00	1.15 – 1.25
	0.13 ± 0.03	1.25 – 1.38
		Total = 1.38 ± 0.11

Table S2: Sampling equipment used for different samples during the cruise

Matrix	Sampling tool
Snow	Aluminium shovel
Sea ice	Kovac ice corer (90 mm internal diameter)
Melt-pond	HDPE Bucket
Under-ice seawater (0.5m)	Niskin bottle
Under-ice seawater (5.0m)	Niskin bottle

Table S3: Density measurements (mean ± 1.s.d.) of different ice type gathered in this study

Compartment	Snow ($n = 7$)	Snow-ice ($n = 7$)	Bulk ice ($n = 17$)
Density ($kg\ m^{-3}$)	180 ± 14	551 ± 84	892 ± 210

The density ($kg\ m^{-3}$) of snow is given by: $$\rho_{snow} = m / V \quad (1)$$

Where m is the measured mass using gravimetric analysis of a snow sample (kg); V is the volume (m^{-3}) of the snow sample taken using a sampling apparatus. In this case, the reference sampling volume was 0.25 dm^3

The density ($kg\ m^{-3}$) of sea ice (snow-ice/bulk ice) is given by: $$\rho_{sea\ ice} = m / \pi \times r^2 \times h \quad (2)$$

Where m is the gravimetrically measured mass (kg) of a sea ice sample shown to follow characteristics representative of a particular ice type (e.g. snow-ice; see Figure 2 in main text); π is 3.142; r is the radius of sea ice corer (0.045 m); h is the sea ice sample length. Due to the method being implemented the measurement precision on sea ice samples (snow-ice and bulk ice) is expected to be slightly less. The density of superimposed ice was not assessed but assumed to fall within the range of bulk ice.

Table S4: Information obtained from the data logger onboard the *Kronprins Haakon* at P6

Latitude	Longitude	Depth (m)	Heading (Degree)	Speed (knots)	Water temperature (°C)	Wind Speed (Knots)	Wind direction (Degrees)	Air temperature (°C)	Air pressure (hPa)	Humidity (%)
8118.708741 N	03121.021065 E	188.46	344.61	0.4	-1.7	5	128	-0.7	999.7	99
8118.780273 N	03119.421987 E	204.34	344.85	0.4	-1.7	4.8	132	-0.8	999.8	100
8122.935053 N	03117.393097 E	0	313.97	1.6	-1.7	4.1	133	0	1000.1	99
8123.085928 N	03116.334402 E	187.3	351.66	0.6	-1.7	4	135	-0.7	1000.1	101
8124.662287 N	03114.735885 E	205.56	319.14	1.6	-1.7	4.6	133	-0.3	1000.3	99
8124.862982 N	03114.295916 E	209.53	321.56	0.5	-1.5	4.5	137	-0.7	1000.2	101
8125.865978 N	03108.691454 E	256.17	274.09	0.5	-1.7	4.4	147	1	1000.4	99
8126.101201 N	03108.976667 E	285.32	268.58	0.5	-1.6	4.7	167	2.3	1000.6	86
8127.560866 N	03104.672063 E	496.41	28.16	0.8	-1.7	4.8	164	-0.5	1000.8	98
8127.832758 N	03105.628658 E	509.32	33.12	0.5	-1.6	4.2	149	-0.8	1001	101
8127.969840 N	03106.359671 E	514.06	33.4	0.5	-1.6	4.2	152	-0.5	1001.1	100
8130.150125 N	03057.533836 E	693.98	17.05	0.6	-1.7	4.6	153	-0.5	1001.2	94
8130.242814 N	03059.397176 E	693.37	20.71	0.4	-1.6	4.7	156	-0.6	1001.5	100
8131.784129 N	03057.330501 E	789.23	349.72	0.1	-1.6	4.6	217	1.6	1003.2	82
8132.487706 N	03056.853998 E	826.2	356.32	0.3	-1.6	5.6	185	0.5	1004.4	82
8132.990799 N	03057.532873 E	865.44	358.28	0.4	-1.7	5.5	172	-2	1004.8	95

Seawater and air temperature measurements were also made hourly via the on-board data logger at sampling station P6

Table S5: Information obtained from the data logger onboard the *Kronprins Haakon* at P7

Latitude	Longitude	Depth (m)	Heading (Degree)	Speed (knots)	Water temperature (°C)	Wind Speed (Knots)	Wind direction (Degrees)	Air temperature (°C)	Air pressure (hPa)	Humidity (%)
8141.135236 N	03033.760504 E	2830.02	298.16	0.1	-1.6	5.4	56	-4	1012.5	99
8159.093220 N	02959.223986 E	3272.97	164.57	0.2	-1.7	6.4	50	-1.1	1012.1	84
8159.047348 N	02958.664982 E	3272.59	164.48	0.3	-1.7	6.1	51	-1.2	1012	83
8159.016541 N	02958.171317 E	3269.47	164.37	0.3	-1.7	7.4	44	-0.8	1011.5	78
8159.003577 N	02957.936389 E	3270.56	164.35	0.3	-1.7	5.9	53	-0.9	1011.7	81
8158.963006 N	02956.623841 E	3274.07	163.83	0.3	-1.7	6.9	36	-0.5	1011.5	83
8158.929513 N	02953.517375 E	3282	162.92	0.4	-1.7	8.9	47	-0.7	1010.7	86
8158.913733 N	02948.027357 E	3290.5	161.88	0.3	-1.7	8	42	-0.4	1009.9	92
8158.918212 N	02947.652112 E	3290.87	161.82	0.3	-1.7	8.5	39	0.2	1009.7	89
8158.879795 N	02944.011224 E	3294.78	160.97	0.2	-1.7	10.3	44	0.6	1008.4	93
8158.867516 N	02943.724824 E	0	160.84	0.3	-1.7	10.4	43	0.8	1008.2	92
8158.175689 N	02937.467284 E	3293.38	160.71	0.5	-1.7	13.7	42	0.2	1005.8	93
8158.160667 N	02937.305832 E	3293.25	160.76	0.5	-1.7	13.2	40	0.1	1005.8	93
8157.071563 N	02918.895781 E	3306.91	162.92	0.5	-1.7	12.8	29	0.7	1002.3	92
8157.054958 N	02918.444469 E	3306.9	162.85	0.5	-1.7	14.7	24	0.6	1002.4	92
8157.024527 N	02917.667809 E	3309.49	162.85	0.5	-1.7	14.3	23	0.8	1002.3	92
8157.002660 N	02917.150975 E	3313.34	162.83	0.5	-1.7	11.8	22	0.6	1002.2	92
8156.980721 N	02916.671655 E	3313.25	162.88	0.5	-1.7	14.5	23	0.7	1002.1	93
8156.965258 N	02916.377383 E	3315.21	162.89	0.5	-1.7	12.9	21	0.7	1002	92

Seawater and air temperature measurements were also made hourly via the on-board data logger at sampling station.

Table S6: Overview of analytical standards, CAS numbers, the standard suppliers, purity and concentration/amount

Acronym	Analytical standard	CAS number	Supplier, purity and concentration/amount
PFBA	perfluoro-*n*-butanoic acid	375-22-4 (acid)	PFC-MXA (mixture) Wellington Laboratories, > 98 % 2.0 µg/mL ± 5 % of the single compounds
PFPeA	perfluoro-*n*-pentanoic acid	2706-90-3 (acid)	
PFHxA	perfluoro-*n*-hexanoic acid	307-24-4 (acid)	
PFHpA	perfluoro-*n*-heptanoic acid	375-85-9 (acid)	
PFOA	perfluoro-*n*-octanoic acid	335-67-1 (acid)	
PFNA	perfluoro-*n*-nonanoic acid	375-95-1 (acid)	
PFDA	perfluoro-*n*-decanoic acid	335-76-2 (acid)	
PFUnDA	perfluoro-*n*-undecanoic acid	2058-94-8 (acid)	
PFDoDA	perfluoro-*n*-dodecanoic acid	307-55-1 (acid)	
PFTrDA	perfluoro-*n*-tridecanoic acid	72629-94-8 (acid)	
PFTeDA	perfluoro-n-tetradecanoic acid	376-06-7 (acid)	
PFBS	potassium perfluoro-*n*-butanesulfonate	29420-49-3 (K+ salt) 375-73-5 (acid)	PFS-MXA (mixture) Wellington Laboratories, > 98 % 2.0 µg/mL ± 5 % of the single compounds
PFHxS	sodium perfluoro-*n*-hexanesulfonate	82382-12-5 (Na+ salt) 355-46-4 (acid)	
PFHpS	sodium perfluoro-*n*-heptanesulfonate	22767-50-6 (Na+ salt) 375-92-8 (acid)	
PFOS	sodium perfluoro-*n*-octanesulfonate	4021-47-0 (Na+ salt) 1763-23-1 (acid)	
PFDS	sodium perfluoro-*n*-decanesulfonate	2806-15-7 (Na+ salt) 335-77-3 (acid)	
13C4-PFBA	perfluoro-*n*-[13C4]-butanoic acid	-	MPFAC-MXA (mixture) Wellington Laboratories, > 98 %, 2.0 µg/mL ± 5 % of the single compounds
13C2-PFHxA	perfluoro-*n*-[1,2-13C2]-hexanoic acid	-	
13C4-PFOA	perfluoro-*n*-[1,2,3,4-13C4]-octanoic acid	-	
13C5-PFNA	perfluoro-*n*-[1,2,3,4,5-13C5]-nonanoic acid	-	
13C2-PFDA	perfluoro-*n*-[1,2-13C2]-decanoic acid	-	
13C2-PFUnDA	perfluoro-*n*-[1,2-13C2]-undecanoic acid	-	
13C2-PFDoDA	perfluoro-*n*-[1,2-13C2]-dodecanoic acid	-	
18O2-PFHxS	sodium perfluorohexane-*n*-[18O2]-sulfonate	-	
13C4-PFOS	sodium perfluoro-*n*-[1,2,3,4-13C4]-octanesulfonate	-	
13C8-PFOA	perfluoro-[13C8]-octanoic acid (injection standard)	-	Wellington Laboratories, > 98 %, (50 ± 2.5) µg/mL

Target PFAS include 11 PFCA (C_4 to C_{14}) and five PFSA (C_4, C_6, C_7, C_8, C_{10})

Table S7: Quality assurance and quality control

Parameter	PFBA	PFPeA	PFHxA	PFHpA	PFOA	PFNA	PFDA	PFUnDA	PFDoDA	PFTrDA	PFTeDA	PFBS	PFHxS	PFHpS	PFOS	PFDS
Laboratory blanks (n=5) (pg L^{-1})	331 ± 108	1 ± 3	41 ± 11	11 ± 4	34 ± 3	1 ± 3	1 ± 3	1 ± 2	1 ± 2	<LOD	<LOD	123 ± 47	<LOD	<LOD	<LOD	<LOD
Field blanks (n=2) (pg L^{-1})	277 ± 74	4 ± 6	38 ± 14	15 ± 6	32 ± 2	3 ± 4	3 ± 4	4 ± 1	<LOD	<LOD	<LOD	67 ± 44	<LOD	<LOD	<LOD	<LOD
*Procedural blanks (n=7) (pg L^{-1})	309 ± 90	2 ± 4	40 ± 11	12 ± 4	33 ± 2	2 ± 3	2 ± 3	1 ± 2	1 ± 2	<LOD	<LOD	107 ± 50	<LOD	<LOD	<LOD	<LOD
Method detection limit (pg L^{-1})	579	13	72	25	41	12	11	7	6	1[†]	4[†]	257	1[†]	1[†]	5[†]	1[†]
Internal Standard (IS)	$^{13}C_4$-PFBA		$^{13}C_2$-PFHxA	$^{13}C_4$-PFOA		$^{13}C_5$-PFNA	$^{13}C_2$-PFDA	$^{13}C_2$-PFUnDA	$^{13}C_2$-PFDoDA			$^{18}O_2$-PFHxS			$^{13}C_4$-PFOS	

*Procedural blanks (n=7) include laboratory (n=5) and field blanks (n=2) and were all used to calculate method detection limits for each PFAA. A mass-labelled analogue of some PFAS was not available (e.g. PFHpA) and therefore a structurally similar chemical was utilised. †Some analytes were not detected in procedural blanks and therefore method detection limits were determined from the signal obtained from the lowest detectable calibration standard with the mean laboratory blank volume (0.8 L).

The method detection limit (pg L^{-1}) is given by:

$$p_{\text{method}} = \bar{x}_{\text{procedural blanks}} + 3.\sigma_{\text{procedural blanks}} \quad (3)$$

where $\bar{x}_{\text{procedural blanks}}$ is the mean of the laboratory and field blanks; $\sigma_{\text{procedural blanks}}$ is the standard deviation of the laboratory and field blanks.

Water mass equivalent of ice per unit area of sea ice (kg m^{-2}) is given by: $$w_n = L_n \times \rho_n \quad (4)$$

where L is the depth (m) of a particular ice sample type; ρ_n is the mean sample density (kg m^{-3}) of a particular type of ice (see Equations S1 – S2).

Mass of PFAA per unit area (pg m^{-2}) of ice is given by: $$q_n = c_{PFAA} \times w_n \quad (5)$$

where c_{PFAA} is the concentration (pg L^{-1}; where 1 L = 1 kg) of an individual PFAA in a particular sample; w_n is the water mass equivalent per unit area of ice (kg m^{-2}).

Volumetrically-weighted concentration of PFAA (pg kg^{-1}) in ice is given by: $$k_n = \Sigma q_n / \Sigma w_n \quad (6)$$

where Σq_n is the total mass of an individual PFAA per unit area (pg m^{-2}) in all samples containing the same ice type (e.g. snow-ice, bulk ice, etc); Σw_n is the total water mass equivalent per unit area (kg m^{-2}) for the same corresponding sample ice type (e.g. snow-ice, bulk ice etc). The volumetrically-weighted concentration of PFAA (pg kg^{-1}) for the entire sea ice system is given by summing the vertical mass of PFAA in the different samples (i.e. snow-ice + bulk ice + superimposed ice) divided by the total sea ice water mass equivalent (i.e. snow-ice + bulk ice + superimposed ice). Volumetrically-weighted $\delta^{18}O$ values in sea ice are also calculated in this manner by exchanging PFAA concentrations (e.g. c_{PFAA}) for $\delta^{18}O$ values (e.g. permil ‰).

PFAA mass fraction (% mass/total mass) in sea ice is given by: $$f_n = q_n / \Sigma q_n \quad (7)$$

where q_n is the total mass of an individual PFAA within a specific ice type (e.g. snow-ice) per unit area (pg m^{-2}); Σq_n, is the total mass of an individual PFAA within the combined types of ice (e.g. snow + snow-ice + bulk ice + superimposed ice) per unit area (pg m^{-2}).

Table S8: Concentrations of PFAA (pg L^{-1}) in sea ice at P6

Sea ice depth (m)	PFBA	PFPeA	PFHxA	PFHpA	PFOA	PFNA	PFDA	PFUnDA	PFDoDA	PFTrDA	PFTeDA	PFBS	PFOS	$\Sigma PFAA_{short-chain}$	$\Sigma PFAA_{long-chain}$
0.00 – 0.10	2259 ± 1184	12 ± 16	29 ± 41	119 ± 79	60 ± 27	86 ± 40	90 ± 52	68 ± 47	28 ± 21	18 ± 16	4 ± 5	186 ± 263	66 ± 13	2604 ± 1577	419 ± 220
0.10 – 0.30	231 ± 326	<MDL	<MDL	88 ± 53	42 ± 25	38 ± 14	39 ± 25	17 ± 8	11 ± 8	7 ± 7	3 ± 5	<MDL	16 ± 23	318 ± 379	174 ± 114
0.30 – 0.50	<MDL	<MDL	<MDL	94 ± 44	8 ± 12	22 ± 12	9 ± 13	6 ± 8	3 ± 5	7 ± 5	<MDL	<MDL	<MDL	94 ± 44	56 ± 55
0.50 – 0.70	<MDL	11 ± 19	<MDL	40 ± 37	8 ± 7	15 ± 1	12 ± 12	8 ± 3	5 ± 8	4 ± 4	<MDL	<MDL	11 ± 10	51 ± 56	61 ± 45
*0.70 – 0.90	<MDL	<MDL	<MDL	256	44	34	54	14	19	7	<MDL	<MDL	<MDL	256	171
0.90 – 1.10	<MDL	<MDL	14 ± 25	112 ± 20	39 ± 34	32 ± 23	22 ± 16	18 ± 13	11 ± 12	1 ± 2	<MDL	<MDL	5 ± 9	126 ± 45	128 ± 109
1.10 – 1.20	232 ± 328	<MDL	62 ± 88	34 ± 48	107 ± 21	51 ± 1	59 ± 15	22 ± 3	24 ± 9	3 ± 4	3 ± 5	<MDL	30 ± 13	328 ± 464	300 ± 70

* sample replicate was not analysed due to technical issue with sample preparation

Table S9: Concentrations of PFAA (pg L^{-1}) in sea ice at P7

Sea ice depth (m)	PFBA	PFPeA	PFHxA	PFHpA	PFOA	PFNA	PFDA	PFUnDA	PFDoDA	PFTrDA	PFTeDA	PFBS	PFOS	$\Sigma PFAA_{short-chain}$	$\Sigma PFAA_{long-chain}$
0.00 – 0.10	7763 ± 9867	161 ± 173	513 ± 594	316 ± 230	815 ± 990	134 ± 37	347 ± 318	132 ± 28	95 ± 83	7 ± 0	6 ± 8	7970 ± 10791	32 ± 12	16723 ± 21654	1568 ± 1476
0.10 – 0.25	5660 ± 7278	93 ± 97	296 ± 418	95 ± 41	527 ± 626	79 ± 3	251 ± 203	53 ± 28	30 ± 11	2 ± 3	<MDL	6578 ± 8770	19 ± 3	12741 ± 16603	960 ± 877
0.25 – 0.43	2548 ± 2711	52 ± 46	137 ± 148	63 ± 14	212 ± 14	48 ± 21	114 ± 101	33 ± 35	54 ± 61	1 ± 1	<MDL	2479 ± 2995	4 ± 5	5280 ± 5914	466 ± 429
0.43 – 0.60	1492 ± 1055	37 ± 17	94 ± 64	51 ±11	144 ± 95	39 ± 7	64 ± 42	11 ± 12	14 ± 17	1 ± 2	<MDL	988 ± 837	8 ± 11	2662 ± 1984	281 ± 185
0.60 – 0.75	593 ± 699	16 ± 14	22 ± 38	62 ± 32	76 ± 40	21 ± 4	34 ± 23	2 ± 4	6 ± 5	<MDL	<MDL	639 ± 434	7 ± 6	1332 ± 1218	146 ± 83
0.75 – 0.90	707 ± 249	20 ± 4	40 ± 7	38 ± 12	93 ± 23	21 ± 0	49 ± 7	<MDL	7 ± 0	<MDL	<MDL	537 ± 323	5 ± 7	1342 ± 595	173 ± 38
0.90 – 1.05	157 ± 223	<MDL	<MDL	23 ± 7	64 ± 5	16 ± 1	37 ± 0	<MDL	<MDL	<MDL	<MDL	323 ± 10	4 ± 6	504 ± 239	121 ± 12
1.05 – 1.15	578 ± 38	41 ± 34	42 ± 59	56 ± 34	87 ± 19	33 ± 12	49 ± 10	8 ± 12	17 ± 15	1 ± 1	0 ± 1	563 ± 187	<MDL	1279 ± 352	196 ± 69
1.15 – 1.25	521 ± 270	25 ± 3	54 ± 22	59 ± 18	79 ± 49	24 ± 2	43 ± 24	<MDL	4 ± 6	<MDL	<MDL	541 ± 470	6 ± 8	1199 ± 782	157 ± 89
1.25 – 1.38	590 ± 438	29 ± 22	38 ± 53	67 ± 10	80 ± 48	53 ± 12	43 ± 18	3 ± 5	14 ± 19	<MDL	<MDL	482 ± 682	15 ± 6	1196 ± 1205	208 ± 108

Table S10: Concentrations of PFAA (pg L^{-1}) in different compartments at P6

Compartment	PFBA	PFBS	PFPeA	PFHxA	PFHpA	PFOA	PFOS	PFNA	PFDA	PFUnDA	PFDoDA	PFTrDA	PFTeDA	$\Sigma PFAA_{short\text{-}chain}$	$\Sigma PFAA_{long\text{-}chain}$
Snow ($n = 3$)	2903 ± 927	<MDL	113 ± 18	110 ± 23	178 ± 100	55 ± 9	<MDL	81 ± 7	28 ± 7	25 ± 11	21 ± 26	<MDL	<MDL	3304 ± 1069	210 ± 61
Sea ice ($n = 14$)	389 ± 874	27 ± 99	4 ± 10	16 ± 36	89 ± 69	42 ± 38	18 ± 24	38 ± 29	38 ± 34	22 ± 25	14 ± 12	6 ± 8	1 ± 3	525 ± 1089	180 ± 174
Seawater (0.5m) ($n = 3$)	<MDL	<MDL	<MDL	128 ± 39	491 ± 214	928 ± 617	42 ± 73	224 ± 118	563 ± 296	128 ± 62	151 ± 100	26 ± 17	37 ± 29	619 ± 254	2099 ± 1313
Seawater (5.0m) ($n = 3$)	<MDL	139 ± 242	<MDL	14 ± 25	144 ± 34	194 ± 39	17 ± 29	80 ± 19	138 ± 17	29 ± 4	37 ± 11	1 ± 2	5 ± 4	298 ± 300	500 ± 127
Melt-pond ($n = 3$)	747 ± 171	<MDL	60 ± 13	61 ± 11	109 ± 34	45 ± 14	<MDL	47 ± 17	3 ± 6	7 ± 7	11 ± 10	<MDL	<MDL	977 ± 228	114 ± 54

Table S21: Concentrations of PFAA (pg L^{-1}) in different compartments at P7

Compartment	PFBA	PFBS	PFPeA	PFHxA	PFHpA	PFOA	PFOS	PFNA	PFDA	PFUnDA	PFDoDA	PFTrDA	PFTeDA	$\Sigma PFAA_{short\text{-}chain}$	$\Sigma PFAA_{long\text{-}chain}$
Snow ($n = 3$)	2356 ± 400	<MDL	63 ± 9	30 ± 26	72 ± 15	27 ± 6	<MDL	47 ± 5	17 ± 8	16 ± 11	10 ± 18	<MDL	<MDL	2520 ± 449	117 ± 47
Sea ice ($n = 20$)	2081 ± 3841	2122 ± 4266	48 ± 66	125 ± 233	82 ± 99	219 ± 368	10 ± 10	47 ± 37	104 ± 139	24 ± 43	24 ± 38	1 ± 2	1 ± 3	4458 ± 8504	430 ± 639
Seawater (0.5m) ($n = 2$)	314 ± 445	<MDL	98 ± 18	16 ± 23	103 ± 49	339 ± 230	15 ± 21	95 ± 30	265 ± 222	25 ± 11	37 ± 16	3 ± 1	4 ± 6	531 ± 535	782 ± 536
Seawater (5.0m) ($n = 2$)	<MDL	<MDL	41 ± 58	<MDL	68 ± 12	107 ± 30	<MDL	41 ± 8	56 ± 24	4 ± 6	7 ± 10	<MDL	<MDL	108 ± 69	214 ± 79
Melt-pond ($n = 3$)	924 ± 151	<MDL	59 ± 21	55 ± 16	131 ± 5	39 ± 7	7 ± 12	44 ± 28	6 ± 6	5 ± 5	10 ± 9	<MDL	<MDL	1169 ± 193	112 ± 66

Table S32: Concentrations of PFAA (pg L^{-1}) in different compartments at P6 and P7

Compartment	PFBA	PFBS	PFPeA	PFHxA	PFHpA	PFOA	PFOS	PFNA	PFDA	PFUnDA	PFDoDA	PFTrDA	PFTeDA	$\Sigma PFAA_{short\text{-}chain}$	$\Sigma PFAA_{long\text{-}chain}$
Snow ($n = 6$)	2629 ± 705	<MDL	88 ± 30	70 ± 49	125 ± 86	41 ± 17	<MDL	64 ± 20	22 ± 9	21 ± 11	15 ± 21	<MDL	<MDL	2912 ± 872	163 ± 78
Sea ice ($n = 34$)	1384 ± 3083	1259 ± 3403	30 ± 55	80 ± 186	85 ± 87	146 ± 294	13 ± 18	43 ± 33	77 ± 112	23 ± 36	20 ± 30	3 ± 6	1 ± 3	2839 ± 6814	327 ± 532
Seawater (0.5m) ($n = 5$)	126 ± 281	<MDL	39 ± 54	83 ± 68	336 ± 262	692 ± 555	31 ± 55	173 ± 111	444 ± 288	87 ± 72	105 ± 95	17 ± 18	24 ± 27	584 ± 666	1573 ± 1220
Seawater (5.0m) ($n = 5$)	<MDL	84 ± 187	16 ± 36	9 ± 19	114 ± 49	159 ± 57	10 ± 23	64 ± 25	105 ± 48	19 ± 14	25 ± 19	1 ± 2	3 ± 4	222 ± 291	386 ± 192
Melt-pond ($n = 6$)	835 ± 174	<MDL	60 ± 15	58 ± 13	120 ± 25	42 ± 10	4 ± 9	46 ± 21	5 ± 5	6 ± 6	10 ± 9	<MDL	<MDL	1073 ± 227	113 ± 59

Concentration data represents mean ± s.d

Table S43: Average sum of PFAA (ng L^{-1}) in different ice types at P6

Ice Type	$\Sigma PFAA_{short-chain}$	$\Sigma PFAA_{long-chain}$
Snow	3.3 ± 1.1	0.2 ± 0.0
Snow-ice	5.1 ± 3.1	0.8 ± 0.4
Bulk ice	0.1 ± 0.2	0.1 ± 0.1
Superimposed ice	n.d	n.d

n.d = not detected

Table S54: Average sum of PFAA (ng L^{-1}) in different ice types at P7

Ice Type	$\Sigma PFAA_{short-chain}$	$\Sigma PFAA_{long-chain}$
Snow	2.5 ± 0.4	0.1 ± 0.0
Snow-ice	10.6 ± 13.3	0.3 ± 0.3
Bulk ice	1.5 ± 0.9	0.2 ± 0.1
Superimposed ice	1.2 ± 1.2	0.2 ± 0.1

Average data represents mean ± s.d

Step 1:

Four sea ice cores were taken at each site

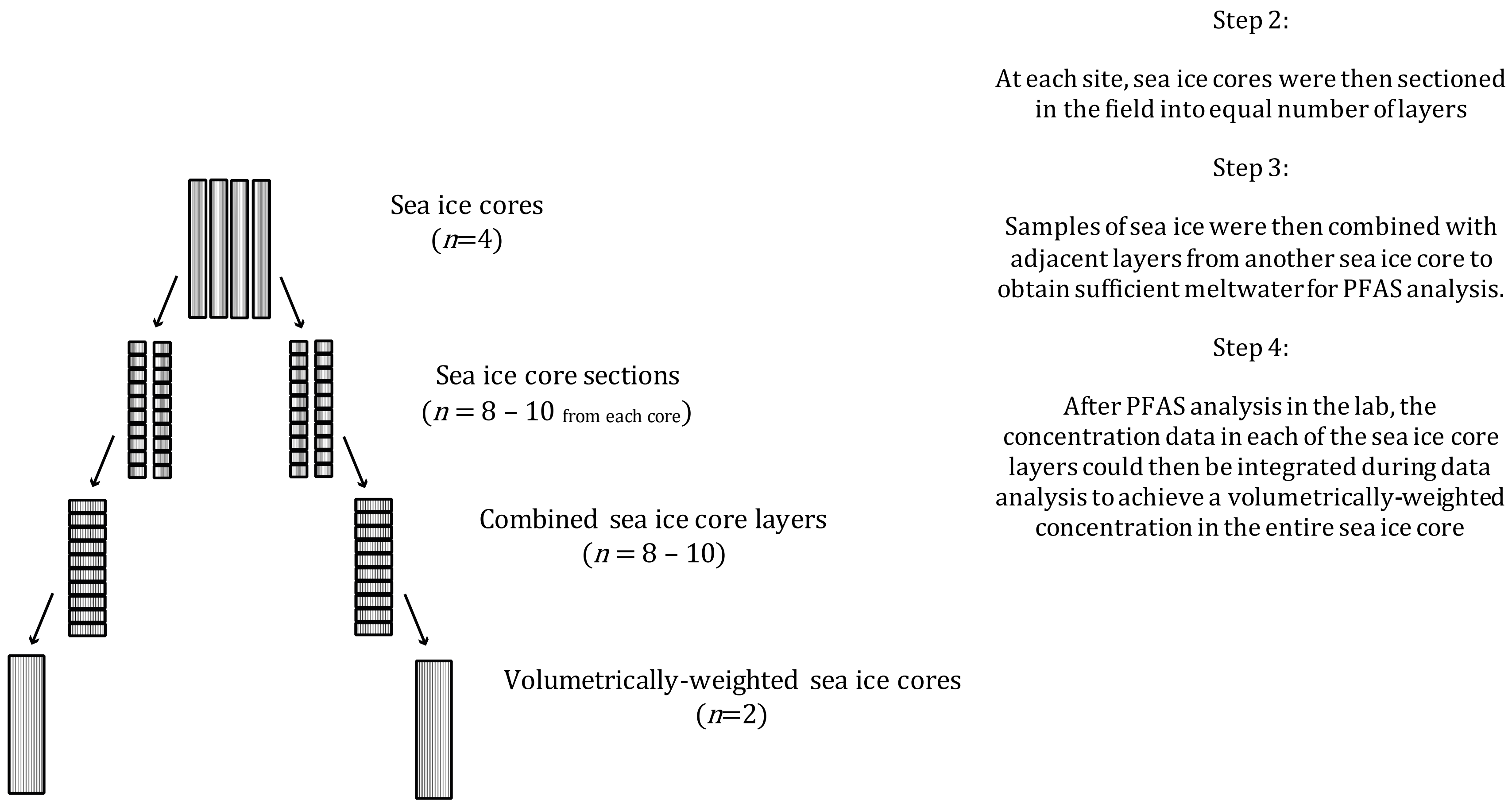

Figure S1: Schematic indicating the sea ice sampling and handling procedures at each site.

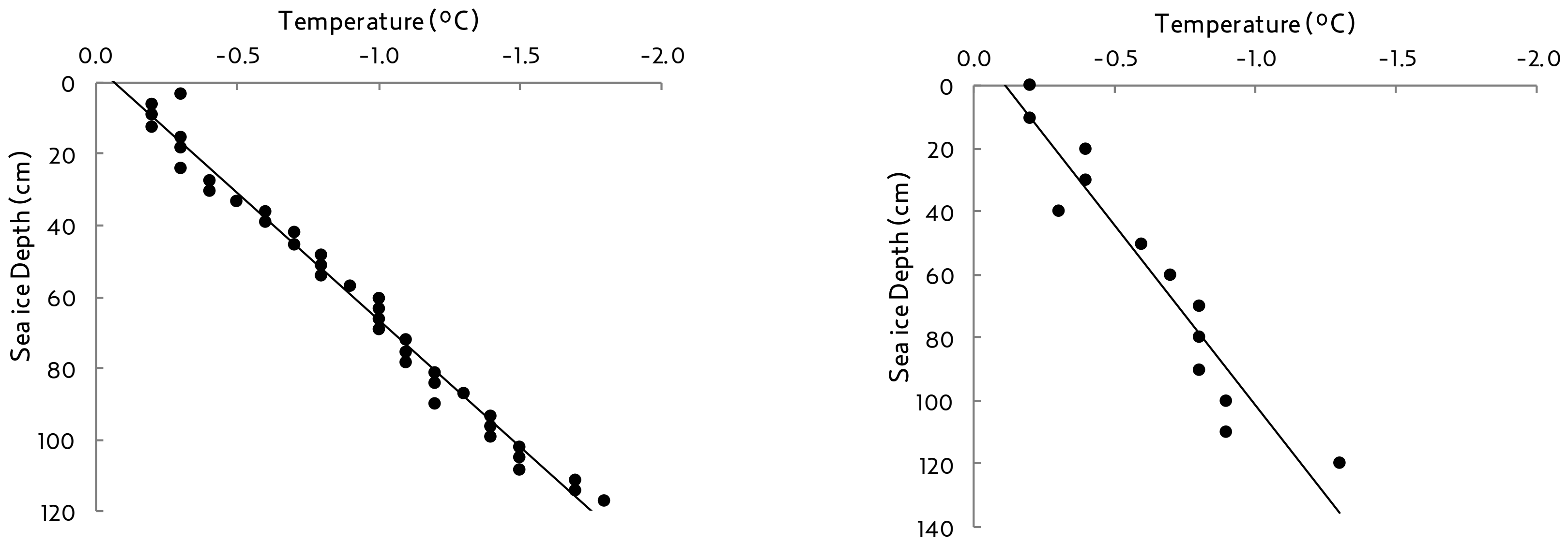

Figure S2: Temperature of sea ice at P6 (left) and P7 (right).

The temperature of sea ice was measured on sea ice cores immediately after extraction. Holes (3 mm diameter) were drilled between 3 cm (P6) and 10 cm (P7) intervals along the length of the core and a probe was inserted and the temperature logged. Temperature profiles showed a negative gradient with depth at both sites, meaning that sea ice growth was unlikely and temperatures were high enough to cause some melting. .

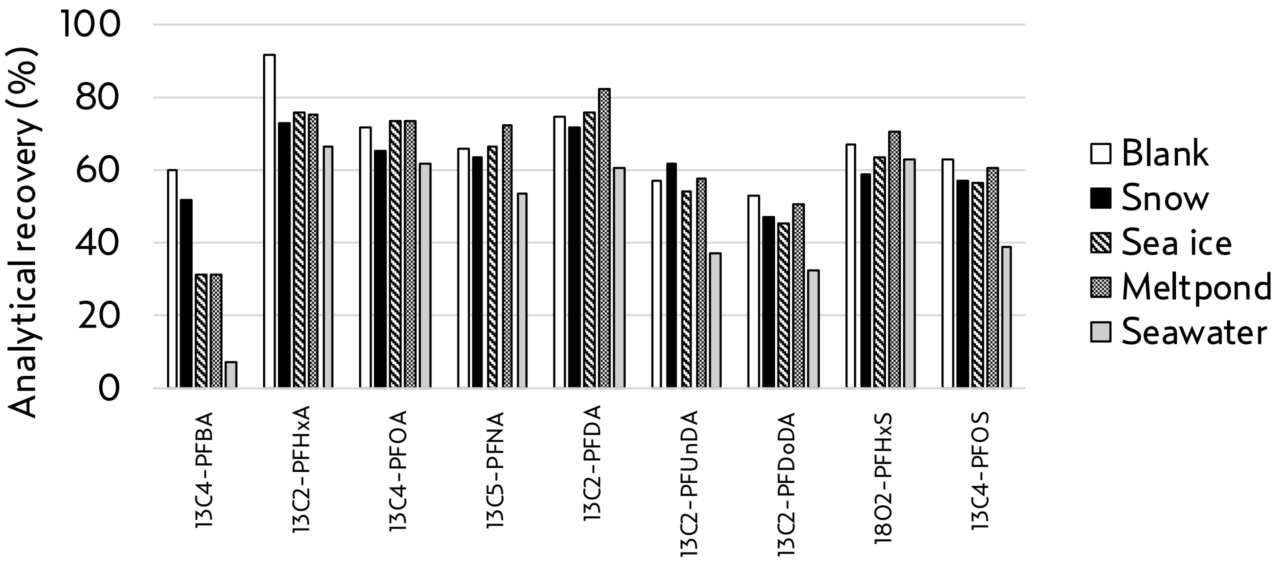

Figure S3 Analytical recovery of surrogate standards in different matrices

Analytical recovery (%) is given by: $$r_{abs} = m_{\text{measured IS,sample}} / m_{\text{actual IS,sample}} \quad (9)$$

where $m_{\text{measured IS,sample}}$ is the mass of internal standard (pg) measured in each sample; $m_{\text{actual IS,sample}}$ is the actual mass of internal standard (pg) introduced into each sample

The absolute analytical recovery (%) of mass-labelled standards was used to monitor the performance of laboratory analytical procedures. In general, recoveries of individual surrogate standards were above 50% which indicated good laboratory methods/procedures. Althoug h, PFBA revealed poor recoveries (<10%) in seawater and was probably linked to matrix effects associated with high levels of salt.

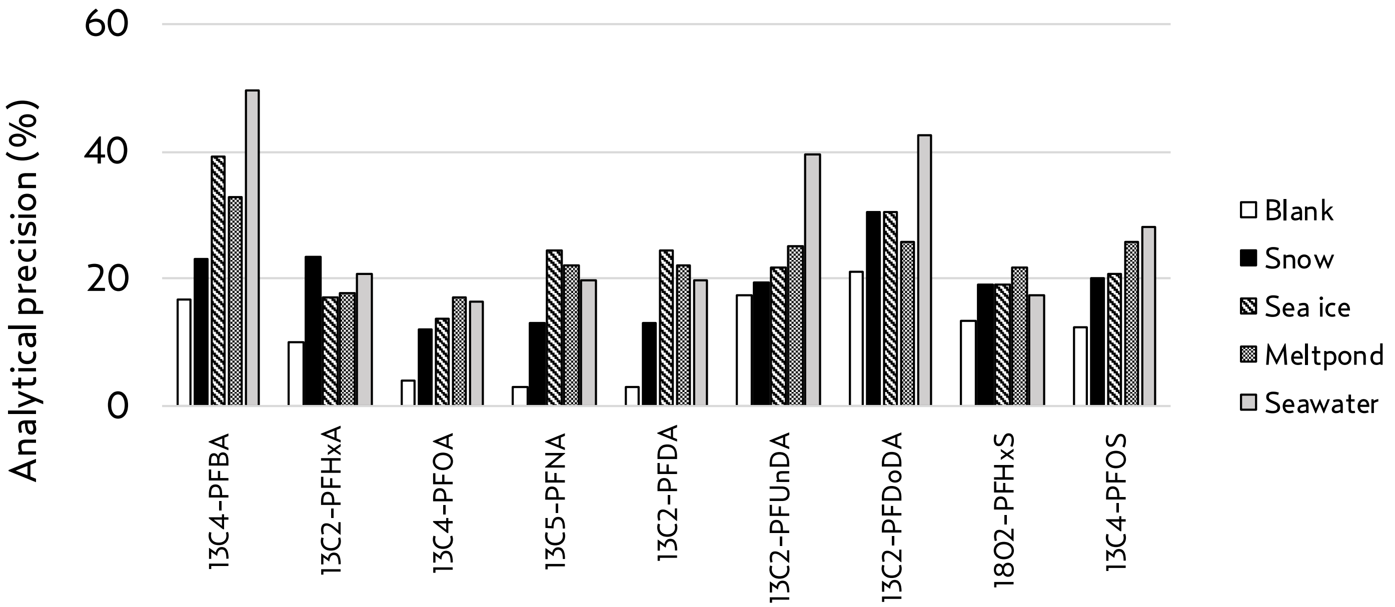

Figure S4: Analytical precision of surrogate standards in different matrices

Analytical precision (%) is given by: $$p_{\text{analytical}} = \sigma_{\text{abs}} / \bar{r}_{\text{abs}} \quad (8)$$

where σ_{abs} is the standard deviation of the absolute recovery for a particular surrogate standard (see Equation S3); $\bar{r}_{abs}$ is the mean of the absolute recovery for a particular surrogate standard

Replicates of field samples were not obtained due to the relatively large sample volume required for PFAS analysis (~1 litre) and therefore the relative standard deviation (RSD) of the absolute recovery was used as an indicator of analytical precision. In general, analytical precision was good (<20%) for individual PFAA, although was higher for some compounds (e.g. $^{13}C_4$-PFBA) particularly in seawater samples. This was probably related to matrix effects.

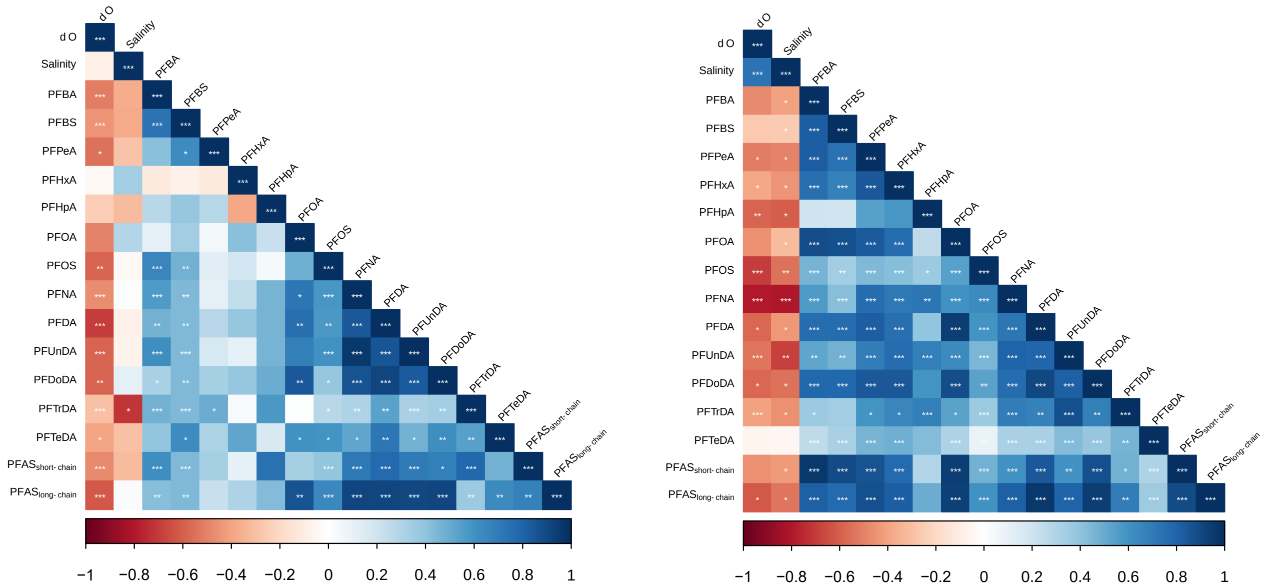

Figure S5: Correlogram of relationships for PFAA, salinity and $\delta^{18}O$ values in sea ice at P6 (left) and P7 (right).

We assessed individual pairwise relationships (Spearmans rank) for PFAA and other physical parameters in sea ice samples at each sampling site. Results showed that most PFAA were positively correlated and were highly significant ($p<0.001$). This indicated that many PFAA share similar sources and behaviour in the Arctic environment. However, short-chain PFAA at P6 showed weak to moderate relationships suggesting they had been strongly influenced by melt processes.

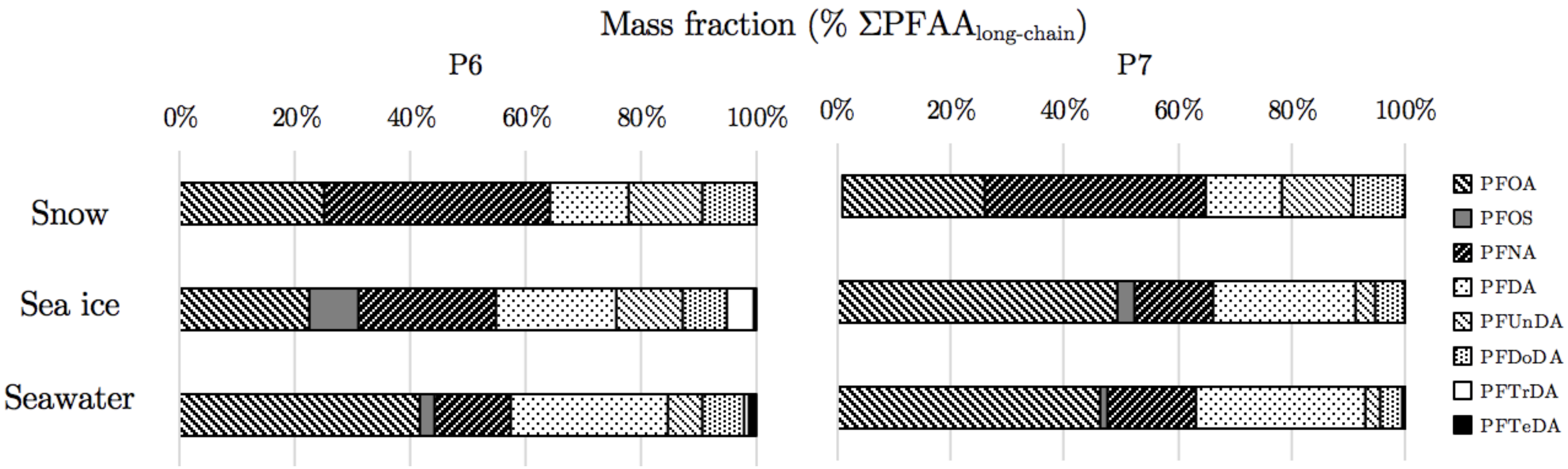

Figure S6: Mass fraction of long-chain PFAA (% $\Sigma PFAA_{long\text{-}chain}$) in different environmental compartments at P6 (left) and P7 (right).

Mass fraction (% $\Sigma PFAA_{long\text{-}chain}$) is given by:

$$f_{PFAA} = c_{PFAA} \quad / \quad c_{\Sigma PFAA} \qquad (10)$$

where c_{PFAA} is the concentration of an individual long-chain PFAA (pg L^{-1}) in a particular sample; $c_{\Sigma PFAA}$ is the sum of long-chain PFAA (pg L^{-1}) in a particular sample.

Mass fraction profiles of long-chain PFAA in sea ice was more comparable to snow than seawater at P6. This indicates that snow had a strong influence on the composition probably through surface melting with subsequent transfer of its PFAA signature into sea ice. In contrast, the mass fraction profile of long-chain PFAA in sea ice at P7 was more comparable to seawater than snow. This suggests that seawater had a dominant effect on the composition, probably via processes related to the initial PFAA uptake from seawater during its formation and through surface flooding of seawater during sea ice ageing.

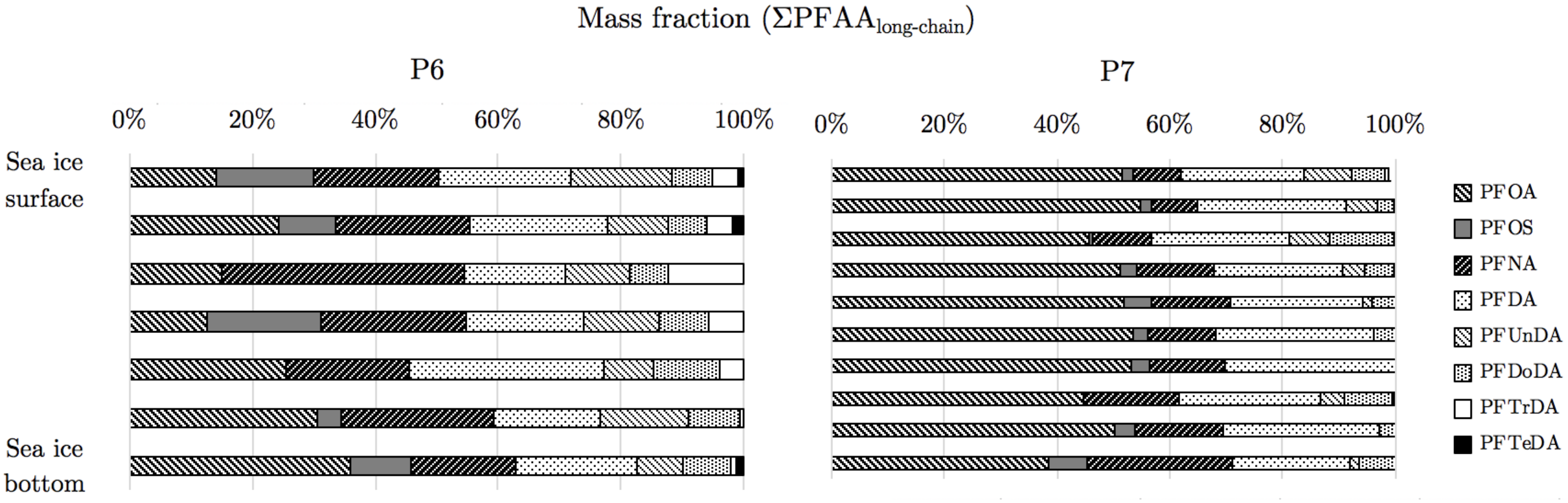

Figure S7: Mass fraction of long-chain PFAA (ΣPFAA$_{long\text{-}chain}$) in sea ice at P6 (left) and P7 (right).

Mass fraction profiles of long-chain PFAA in sea ice was significantly different between P6 and P7, although revealed relatively consistent profiles in the different layers at each site. This suggests that sea ice at P6 and P7 were influenced by separate processes (i.e., snowmelt or surface flooding of sea ice with seawater) resulting in different PFAA compositions.

Table S15: Measured salinity and $\delta^{18}O$ values (mean $\pm$1.s.d) in different end-members

Sample type	$\delta^{18}O$ (‰)	Salinity
Snow	-15.2 $\pm$ 1.1	0.0 $\pm$ 0.0
Seawater	-0.1 $\pm$ 0.4	31.1 $\pm$ 0.7
* Sea ice	0.8 $\pm$ 0.5	2.6 $\pm$ 0.5

Values displayed represent averages of all respective sample types taken at P6 and P7. *Volumetrically-weighted values in sea ice were used to avoid sample bias.

The measured values for the different end-members were then used as constants in the following equations in order to determine the mass fraction in each of the millponds sampled in this study (Marsay, et al., 2018).

$$F_{snow} + F_{sea\ ice} + F_{seawater} = 1 \qquad (11)$$

Where F_n is the mass fraction of end-member (i.e., snow, sea ice ice or seawater) in the meltpond

$$\delta^{18}O_{meltpond} = (F_{snow}\ \delta^{18}O_{snow}) + (F_{sea\ ice}\ \delta^{18}O_{sea\ ice}) + (F_{seawater}\ \delta^{18}O_{seawater}) \qquad (12)$$

Where F_n is the mass fraction of end-member (i.e., snow, sea ice ice or seawater) in the meltpond; $\delta^{18}O_{meltpond}$ is measured $\delta^{18}O$ values (‰) of end-member (i.e., snow, sea ice ice or seawater) in the meltpond.

$$S_{meltpond} = (F_{snow}\ S_{snow}) + (F_{sea\ ice}\ S_{sea\ ice}) + (F_{seawater}\ S_{seawater}) \qquad (13)$$

Where F_n is the mass fraction of end-member (i.e., snow, sea ice ice or seawater) in the meltpond; $S_{meltpond}$ is the measured salinity values of end-member (i.e., snow, sea ice or seawater) in the meltpond.

Equations S11 – S13 were solved simultaneously using the systems of equations calculator (https://www.symbolab.com/solver/system-of-equations-calculator) along with measured salinity/$\delta^{18}O$ values (See Table S16) in each individual meltpond.

Table S16: Measured salinity and $\delta^{18}OH_2O$ values in meltponds at P6 and P7 and their estimated mass fraction contribution of end-members.

Meltpond	Sampling station	Approx. dimensions (Depth x Width x Length)	$\delta^{18}O$ (‰)	Salinity (ppt)	F_{snow}	$F_{seawater}$	$F_{sea\ ice}$
Meltpond 1	P6	0.26 x 3.5 x 1.7	-5.9	2.7	0.42	0.04	0.54
Meltpond 2	P6	0.50 x 3.0 x 7.0	-4.7	1.4	0.34	-0.01	0.67
Meltpond 3	P6	0.18 x 1.0 x 8.0	-3.8	2.0	0.29	0.00	0.71
Meltpond 1	P7	0.30 x 2.0 x 8.0	-2.5	2.1	0.21	0.00	0.79
Meltpond 2	P7	0.34 x 2.0 x 6.0	-6.5	1.3	0.46	0.00	0.55
Meltpond 3	P7	0.38 x 7.0 x 30.0	-5.7	1.0	0.41	-0.02	0.61
					mean (± s.d.) = 0.35 ± 0.09	mean (± s.d.) = 0.00 ± 0.02	mean (± s.d.) = 0.64 ± 0.10

Table S17: Measured PFAA concentrations (mean ± s.d.) in the different end-members.

Measured concentrations (pg L^{-1})	PFBA	PFBS	PFPeA	PFHxA	PFHpA	PFOA	PFOS	PFNA	PFDA	PFUnDA	PFDoDA	PFTrDA	PFTeDA
Snow	2629 ± 705	<MDL	88 ± 30	70 ± 49	125 ± 86	41 ± 17	<MDL	64 ± 20	22 ± 9	21 ± 11	15 ± 21	<MDL	<MDL
Sea ice*	903 ± 1268	812 ± 1404	21 ± 25	52 ± 75	75 ± 41	100 ± 121	9 ± 3	33 ± 11	55 ± 48	15 ± 5	14 ± 11	3 ± 4	1 ± 1
Seawater	63 ± 199	42 ± 132	28 ± 45	46 ± 61	225 ± 213	426 ± 466	21 ± 41	118 ± 95	274 ± 264	53 ± 61	65 ± 77	9 ± 15	13 ± 22
Meltpond	835 ± 174	<MDL	60 ± 15	58 ± 13	120 ± 25	42 ± 10	4 ± 9	46 ± 21	5 ± 5	6 ± 5	10 ± 9	<MDL	<MDL

* Mean volumetrically-weighted values in sea ice were used to avoid possible sample bias originating from differences in the number of sea ice layers taken at either site. Seawater PFAA concentrations include samples acquired at 0.5 m and 5 m depths.

Table S18: Predicted PFAA concentrations (mean ± s.d.) in meltponds based on measured concentrations in end-members and their mass fraction (%) contribution.

Predicted concentration (pg L^{-1})	PFBA	PFBS	PFPeA	PFHxA	PFHpA	PFOA	PFOS	PFNA	PFDA	PFUnDA	PFDoDA	PFTrDA	PFTeDA
Meltpond	1507 ± 1172	523 ± 1179	44 ± 30	58 ± 67	93 ± 60	80 ± 86	6 ± 2	44 ± 17	44 ± 35	17 ± 8	15 ± 15	2 ± 3	0 ± 1
